# Low cost of shifting flight altitude in migrating great snipes

**DOI:** 10.1242/jeb.252022

**Published:** 2026-08-28

**Authors:** Jesper E. Brodersen, Åke Lindström, Thomas Alerstam, Arne Andersson, Johan Bäckman, Anders Hedenström, Julia Weber, Sissel Sjöberg

**Affiliations:** Department of Biology, Lund University, 223 62 Lund, Sweden

**Keywords:** Flight behaviour, Non-stop flight, Climb rates, Sink rates, *Gallinago media*, Bird migration

## Abstract

Many birds make migratory flights lasting several days. Migrants do not only repeatedly make small-scale shifts in altitude; some of them also climb and sink several thousand metres in a diel cycle. This is a seemingly energetically costly behaviour, adding to an already high energetic cost of flight. Here, we describe the climbing and sinking behaviour of migratory great snipes, *Gallinago media*, and discuss whether their diel altitude cycle could represent an additional energy cost to their migration. Making use of tracking data from multisensor data loggers, we quantified altitude variation and compared climb and sink rates from non-stop migratory flights lasting up to 4 days (84 h). In general, great snipes climb and sink at comparatively slow rates, averaging 0.21 m s^−1^ and −0.22 m s^−1^, respectively, almost invariably within ±0.7 m s^−1^, and hardly ever close to a theoretical maximum climb rate of 1.8 m s^−1^. Great snipes do not climb faster at night than in the day and climb and sink rates are similarly low for all long non-stop flights. The additional cost of shifting flight altitude for great snipes is apparently negligible compared with the cost of forward flight (<1%). This is largely explained by the birds flapping during a slow descent, thereby turning most of the potential energy built up during previous climbs into aerodynamic work. We conclude that the overall cost of shifting flight is probably low for most migratory birds, given that climb and sink rates are low.

## Teaser

**Highlighted Article:** During their long non-stop flights, great snipes reduce the total energetic cost of shifting flight altitude to negligible levels by climbing and sinking at low rates.

## INTRODUCTION

The seasonal migration of millions of birds is a spectacular event, but among the masses, some species truly stand out. These are species that push the boundaries of what we think birds are capable of, whether it is an arctic tern, *Sterna paradisaea*, circumventing the globe (Egevang et al., 2010), a bar-tailed godwit, *Limosa lapponica*, flying for almost 10 days non-stop (Gill et al., 2009), or a common swift, *Apus apus*, staying airborne for up to 10 months (Hedenström et al., 2016). However, migratory birds do not only fly far and for long periods; some also fly unexpectedly high. Recently, more attention has been directed towards flight altitude, the third spatial dimension of migratory journeys. The miniaturization of tracking technology has allowed us to ask new questions on how high birds fly and what drives them to these heights, bringing with them surprising new insights.

For instance, it was long assumed that the vertical profile of a bird's flight would be like that of a commercial jet plane. That is, the flight would start with a climb up to a cruising altitude with beneficial winds and then the bird would remain there until the final descent (Hedenström and Alerstam, 1994). The first study to contradict this assumption came from Bowlin et al. (2015), showing that Swainson's thrushes, *Catharus ustulatus*, altered their flight altitude by >100 m repeatedly throughout a migratory flight. Multiple species have since been shown to change flight altitude when migrating (Liechti et al., 2018; Sjöberg et al., 2018) and some at considerably larger scales than the Swainson's thrushes (e.g. Senner et al., 2018; Lindström et al., 2021; Sjöberg et al., 2021). Perhaps the most astonishing performance of them all comes from great snipes, *Gallinago media*, which repeatedly climb to heights above 7000 m above sea level (m.a.s.l.). for reasons that are still not fully understood (Lindström et al., 2021; Sjöberg et al., 2023).

It is well known that migrating birds prefer to fly with favourable tailwinds and that they select times and altitudinal strata with local optima of wind conditions for their flights (Alerstam, 1979; Mateos-Rodríguez and Liechti, 2012). It has also been suggested that birds alter their flight altitude to avoid high temperatures (Senner et al., 2018). One can indeed expect wind conditions and ambient temperature to affect the birds' preferred flight altitude, depending on changing wind and temperature profiles on different occasions (Senner et al., 2018). Sjöberg et al. (2021) found a diel pattern in the climbing and sinking of great reed warblers, *Acrocephalus arundinaceus*. They observed that great reed warblers, primarily night migrants, climbed several kilometres to higher altitudes when they prolonged their flight into the day. If still flying at sunset, they returned to lower flight altitudes. In parallel it was found that great snipes also reached their highest flight altitudes in the day and returned to lower heights in the night (Lindström et al., 2021). As both these species already flew at relatively high altitude during nocturnal flights (average 2000–3000 m), neither winds nor ambient temperature would have changed much between night and day (Wallace and Hartranft, 1969; Seidel et al., 2005) and therefore these are unlikely to have been drivers behind these large altitude shifts. Hence, three hypotheses have been proposed to explain what could be driving diel altitude cycles in migrating birds: increased range of vision, predator avoidance and that the birds seek higher, and thus colder, altitudes to counteract the heating effect of solar radiation combined with the hard work of continuous flapping flight (Lindström et al., 2021; Sjöberg et al., 2021, 2023). It should be noted that even though these hypotheses might explain the observed differences between diurnal and nocturnal flight altitudes during long continuous flight, the hypotheses do not exclude the possibility that birds adjust their flight altitude to gain wind support or to avoid high ambient temperatures within diurnal and nocturnal periods of flight. It is reasonable to expect that the observed large differences in flight altitude between night and day, and the smaller altitude changes within night and day have separate adaptive causes.

So far, it has been implicitly assumed that large altitude shifts by birds migrating by flapping flight pose an additional cost to already energetically demanding migrations, as climbing flight is costly (Hedenström and Alerstam, 1992, 1994). Following this assumption, the selection pressure driving birds to extreme heights in the day should be strong. However, the energetic cost of changing altitude by migrating birds has seldom been analysed in detail, and never for the entirety of a flight (Hedenström and Alerstam, 1992, 1994; Piersma et al., 1997). Climb rates have either been analysed for parts of the initial climb (Hedenström and Alerstam, 1992, 1994; Piersma et al., 1997) or averaged across the whole flight (Sjöberg et al., 2018). One could for example predict that climb rates increase the longer the bird has been flying, as it becomes energetically cheaper when the bird has burned a significant amount of its fuel load, and therefore has a lower body mass, so more power is left for climbing. Such a question can only be answered from analysing climb rates from tracks of complete migratory flights. Further, estimates of the full energetic cost of changes in flight altitude will remain mere guesses until the climbing and sinking behaviour of birds has been described in detail.

**Fig. 1.**
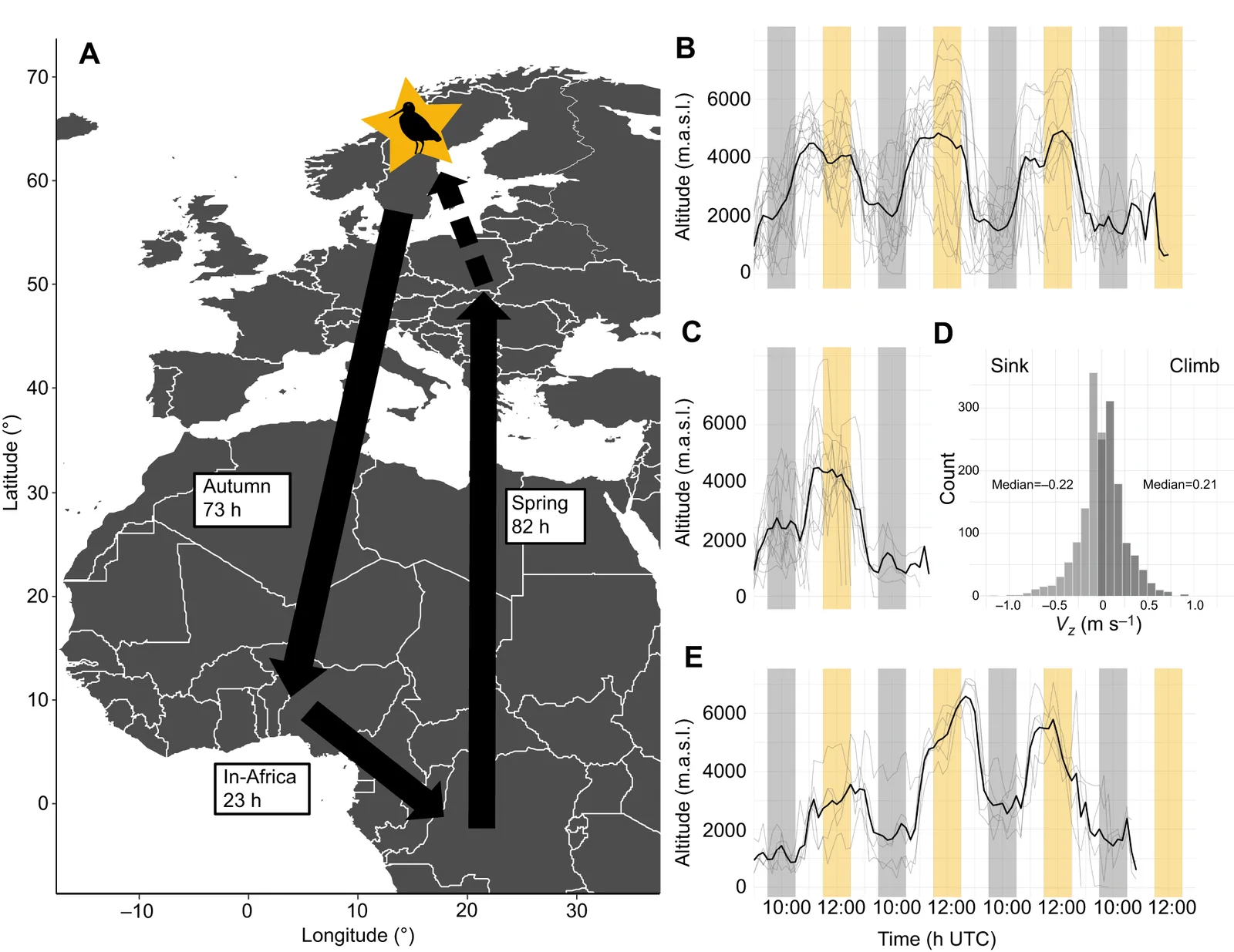
**A summary of great snipe marathon flights throughout their annual migration cycle.** (A) A schematic map of the three marathon flights performed by great snipes from our study site in mid-Sweden; Autumn, in-Africa and Spring. The average durations are shown for each respective flight (Lindström et al., 2021). Autumn (B), in-Africa (C) and spring (E) migration flight curves with hours classified as day (08:00–14:00 h UTC) marked in yellow and hours classified as night (20:00–02:00 h UTC) in grey. Average flight altitude for each hour of flight is shown in black and individual flight curves are plotted in grey. (D) The frequency distribution of climb (dark grey) and sink rates (light grey) for all marathon flights included in the study at a 1 h resolution.

With our study, we aimed to test whether the climbing and sinking behaviour of migrating great snipes is energetically costly, based on data from multisensor data loggers. Great snipes invariably perform three long non-stop flights during their annual migrations to, within and from their sub-Saharan wintering grounds, in addition to several strictly nocturnal shorter flights (Lindström et al., 2021). During the long flights, from now on referred to as ‘marathon flights’, they fly for, on average 73, 23 and 82 h, respectively, and while doing so they repeatedly climb to altitudes >7000 m.a.s.l. (Fig. 1; Lindström et al., 2021).

We addressed three main research questions in this study. First, we investigated how often and at what scale great snipes vary their flight altitude in their non-stop flights. Second, we quantified at what rates (in m s^−1^) great snipes climb and sink during these flights and how these rates may vary with flight altitude and time of day. Third, we provide an estimate of the overall added energetic cost of the total changes in flight altitude of individual flights to answer how costly shifting flight altitude could be for these migratory birds.

When analysing climb and sink rates, we first tested whether the time spent on the wing affects climb or sink rates. The further the snipes progress in their flights, the lower their body mass should become as they consume their fuel supplies. When comparing climb rates of several species, Hedenström and Alerstam (1992) found a negative relationship between climb rate and body mass. If this relationship also holds true within individuals and if climb rate is limited by muscle power, we would expect great snipes to climb faster the longer they have spent on the wing, as climbing will become energetically cheaper later in the flight.

As far as sink rates go, it would make sense for the birds to do this slowly, because in this way they could transfer most of the potential energy built up during previous climbs into the aerodynamic work of forward flight (Pennycuick, 1969, 1975). Jenni-Eiermann et al. (2024) found that birds migrating over continental Europe sink at low rates in the last 1.5 h of their migratory flights. They also found that birds caught late at night, nearing the end of their migratory flight, showed signs of a lowered metabolic intensity measured through blood metabolites. They suggest that the combination of these findings could indicate that birds sink slowly to make maximum use of the potential energy gained during previous climbs. Accordingly, we would expect sink rates to be lowest at the end of great snipe marathon flights.

We then tested whether flight altitude influences the climbing performance of great snipes. The energy cost of flight over distance is expected to remain the same across altitudes, high or low (Pennycuick, 1969). But the flight speed at which this balance is maintained is higher, meaning that a bird must allocate more of its available flight power to forward flight, thereby reducing the power margin left for climbing flight. This reduction in climbing power margin with altitude stems from the increased difficulty in producing lift to counteract the weight and thrust for propulsion with lower air density. A bird can increase the lift its wings produce by generating more thrust to fly faster, but only until it has reached its maximum power output available from the flight muscles. Accordingly, as air density decreases when the bird flies higher, the power margin left for climbing becomes smaller.

The power required to fly at a certain speed varies inversely with the square root of air density, following eqn 51 from Pennycuick (1969). The power margin left for climbing flight should vary proportionately, if the maximum power available for flight does not change. This means that for every 1000 m of elevation gain, a bird will have a 5% smaller power margin left for climbing, under standardized atmospheric conditions (Table 1). For a great snipe climbing at its maximum capacity, we can then predict a 5% decrease in climb rate for every kilometre of altitude gained.

**Table 1.** **Air density, ρ, and the inverse square root of ρ for every 1000 m of elevation gain in a standard atmosphere, modified from (** **Pennycuick, 1975** **)**

Altitude (m.a.s.l.)	ρ (g cm^−3^)	1/√ρ
0	1.22	0.905
1000	1.11	0.949
2000	1	1.000
3000	0.905	1.051
4000	0.813	1.109
5000	0.732	1.169
6000	0.656	1.235

Modified from Pennycuick (1975).

To further explore patterns in the climbing and sinking behaviour of great snipes, we compared climb and sink rates for the three marathon flights and between flight during the day or night. We would expect to see the highest climb rates in the in-Africa flight of great snipes because this is probably the flight in which they take off with the lowest fuel load and therefore have a larger power margin left for climbing. If great snipes climb up to high altitudes in the day to avoid overheating (Sjöberg et al., 2023), we would expect their climbs at these altitudes to be restricted by the added heat load of solar radiation. So, we would predict climbs to be limited to lower rates in the day than in the night. All the above reasoning builds on the assumption that migratory birds regularly climb close to their maximum capacity, which however remains to be investigated.

## MATERIALS AND METHODS

### Field methods

We caught adult male great snipes, *Gallinago media* (Latham 1787), in mist nets at leks in Jämtland, Sweden. We fitted the birds with multisensor data loggers. The loggers were custom made at Lund University (Bäckman et al., 2017) and fitted onto rings put on the birds' tibia. All loggers were equipped with sensors for barometric pressure and *z*-axis acceleration. We used Bosch Sensortec pressure sensors with temperature compensation and an absolute accuracy of ±1 h Pa at 950–1050 h Pa and 0–40°C. The sensors have an accuracy of ±3 h Pa outside this range (300–1100 h Pa, 0–65°C). We derived flight altitude (m.a.s.l.) from barometric pressure, assuming standard atmospheric conditions at sea level (ISO 2533:1975 – Standard Atmosphere). Loggers were programmed to sample activity and pressure either every hour or every 5 min (Bäckman et al., 2017). The loggers sampling activity every 5 min were programmed to start recording pressure at 5 min intervals after a series of three high activity levels (hence not recording barometric pressure throughout the year but only during active flight). From here on, we will refer to the loggers with a 1 h sampling interval as ‘1 h loggers’ and those with a 5 min sampling interval as ‘5 min loggers’. We include a total of 45 flights from 24 individuals in this study. Of these flights, 35 1 h loggers had functional pressure recordings, including 18 autumn, 12 in-Africa and 5 spring flights (Fig. S1). Ten flights contained functional pressure data from 5 min loggers, with 5 autumn, 4 in-Africa and 1 spring flight (Fig. S2). For more information on the multisensor data loggers, see Bäckman et al. (2017) and Sjöberg et al. (2018). For information on how to identify flight from the activity data for great snipes, see Lindström et al. (2021). The loggers analysed were put on birds in 2016–2021 and retrieved one or more years later.

This work was conducted under permits from the Swedish Bird Ringing Centre, the Lund/Malmö Ethical Committee for Animal Experiments (M33-13, M72-15 and 5.8.18-6518/2020) and the Jämtland regional county board to work in Vålådalen nature reserve (522-4141-2013, 522-1294-18, 522-1624-2023).

### Flight altitude shifts – how often and how large?

We studied altitude variability in two different ways: how often great snipes switched altitude and at what scale they did so. As a measure of how often they changed flight altitude, we calculated vertical tortuosity, defined as the number of switches between climbing and sinking flight (or vice versa) divided by the total number of altitude recordings minus 1 (Norevik et al., 2021). Vertical tortuosity thereby ranges between 0, meaning one continuous climb or sink and 1, indicating a change in vertical direction for every altitude recording made. To capture at what scale the snipes changed altitude, we calculated the total change in altitude for each continuous climb or sink segment. From here on, we refer to this value as segment altitude change. We calculated both the median vertical tortuosity and segment altitude change for all flights with functional pressure data in a 1 h temporal resolution. For these analyses, the data from the 5 min loggers were resampled every hour and included in the dataset. If not stated differently, all analyses were done using all available flights at a 1 h temporal resolution.

We defined narrow time windows of day (08:00–14:00 h UTC) and night (20:00–02:00 h UTC) for the flights (Sjöberg et al., 2023). Great snipes might pass through one or two time zones in their annual cycle and encounter variable daylengths. To control for this, we used these narrow definitions of day and night. Any other time of day, including twilight, when the snipes typically undertake large shifts between night-time and daytime altitudes, we labelled as ‘other’.

For the entire dataset, we calculated vertical tortuosity of the combined recordings in each time of day for every flight. Thus, each flight had one value of vertical tortuosity for day, night and ‘other’, respectively. We fitted the data to linear mixed effects models, using *lmer* in the R-package *lme4* (Bates et al., 2015) with time of day (day, night, other) and flight (autumn, in-Africa and spring) as fixed effects. To avoid pseudoreplication, we set bird identity as a random effect. We tested for differences in vertical tortuosity between the fixed effects using a Wald's *F*-test and type three sums of squares.

For each segment of continuous climbing or sinking, we calculated the accumulated amount of vertical change, i.e. segment altitude change. We let the starting time of the segment define what time of day it was categorized as, as this time would reflect the conditions under which the birds chose to shift altitude. We fitted the data to a log-linear mixed effects model with *lmer* in *lme4* (Bates et al., 2015) with time of day and flight as fixed effects and logger identity as a random effect. We used a Wald's *F*-test with type three sums of squares for significance testing of the fixed effects.

### Flight altitude shifts – how fast?

We computed overall median and maximum climb and sink rates for all flights in our dataset, and for the 5 min subset separately. For median vertical speed, we calculated the respective interquartile range (IQR), corresponding to the middle 50% of all values in ascending order. When analysing climb and sink rates, the sampling interval of the logger might influence how much variation is captured in the data. A longer sampling interval might miss the very fastest climbs and sinks, which are potentially short in duration. Therefore, we tested whether climb and sink rates of the 1 h logger subset were statistically different from those for the 5 min subset. The tests were performed using a Mann–Whitney *U*-test, comparing climb and sink rates separately.

We tested the effect of time on the wing on the climb rates of great snipes, to see whether great snipes climbed faster at the end of their marathon flights when climbing costs should be lowest. We fitted climb rates to a log-linear mixed effects model with the time passed since the start of the flight as a fixed effect and logger identity as a random effect. To see whether snipes sink slower the longer they have been flying, we fitted a similar log-linear model to sink rates, using the same fixed and random effects as above. In this model, we only included tracks of autumn and spring flights that were similar in length. To test for statistical significance, we used a Wald's *F*-test.

To test the relationship between climbing performance and flight altitude, we made use of the data subset with a 5 min sampling interval. The reason for this is that a bird climbing at 1 m s^−1^ for 1 h will gain 3600 m of elevation. At high altitudes, such a climb rate is highly unlikely to be found, meaning that a negative relationship between climb rate and altitude is to be expected by default and would not reflect the climbing capacity of the bird. By using our data with a 5 min sampling interval, we minimized this effect. We first fitted climb rates faster than 0.1 m s^−1^ against flight altitude to a linear mixed effects model. The cut-off value of 0.1 m s^−1^ was chosen arbitrarily to exclude ‘climbs’ which were close to negligible and may even reflect unintended changes in flight altitude. To test the same relationship for maximum climb rates, we extracted the maximum climb rate observed for each 100 m of altitude interval and fitted these values to a linear mixed effects model. For both models, we tested for significance with a Wald's *F*-test.

We compared climb and sink rates for day and night and between the three marathon flights. We fitted climb and sink rates to separate log-linear mixed effects models, with time of day and flight as fixed effects and logger identity as a random effect. We tested for significance using a Wald *F*-test, computed the estimated marginal means and performed a pairwise contrast analysis with the R-package *emmeans* (https://CRAN.R-project.org/package=emmeans)*.* For this second analysis, we resampled the 10 flights with a 5 min sampling interval to an hourly temporal resolution, to make use of the full dataset.

Third, we tested whether the length of the climb or sink segment influences how fast great snipes climb or sink. We calculated the median vertical speed and the length of each continuous climb or sink segment in both the 1 h and 5 min datasets. Then, we fitted climb and sink rates of these segments to respective log-linear mixed effects models with segment duration as a fixed effect and logger identity as a random effect. We used a Wald *F*-test to test for statistical significance of the fixed effect.

We investigated whether great snipes ever climb at their theoretical maximum climb rate during their migratory flights. For this analysis, we chose to only include data from our 5 min loggers, so as not to lose detail, given that we expect the fastest climbs to be performed over short durations. This also enabled comparison with another study calculating maximum climb rate at a 5 min sampling interval (Norevik et al., 2021). We used the function *computeFlightPerformance* in the R-package *afpt* (Klein Heerenbrink et al., 2015; https://CRAN.R-project.org/package=afpt) to calculate the maximum climb rate for a great snipe with the standard wing measurement given in the package. To avoid underestimating the maximum climb rate, we here assumed a body mass of 160 g, roughly corresponding to a male's lean body mass (Cramp and Simmons, 1983). Using a one-sample Mann–Whitney *U*-test, we tested whether the maximum climb rate performed by migrating great snipes differed from the theoretical maximum climb rate. It should be noted that the 5 min sampling interval subset contains two outlier climb rates of 3.45 and 2.86 m s^−1^. These incredibly fast climbs occurred at the end of a single autumn flight (X8BD_AUT: see Fig. S3) in a series of rapid ascents and descents. As we cannot confirm whether these climb rates are correct or instead arose from temporary sensor malfunction, we chose to perform the test both with and without them. They were included in the previous analysis using the 5 min subset but do not affect our conclusion there.

All analyses in our study were performed in R, version 4.5.0 (https://www.r-project.org/). Throughout the analysis, we adopt a significance level of 0.05 where effects below this level were considered statistically significant.

### Flight altitude shifts – how costly?

**Fig. 2.**
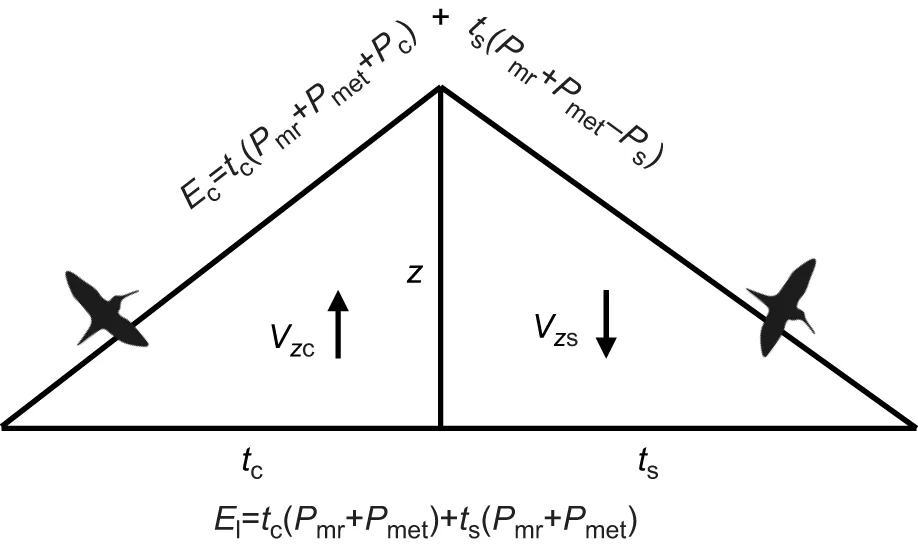
**A simple model for estimating the energetic cost of a bird's total shifts in flight altitude compared with a hypothetical horizontal flight.** *E*_c_ is the energy required for the flight, including the cumulative height gained during the climbing and sinking phases (*z*). *E*_l_ is the energy required for the flight if it had been horizontal. The time spent climbing is written as *t*_c_ and the time spent sinking as *t*_s_. *V_z_*_c_ denotes the median climb rate of the flight and *V_z_*_s_ corresponds to the median sink rate of the flight. *P*_mr_ is the power to sustain flight at maximum range speed, *P*_met_ is the power needed for the bird's basal metabolic rate, *P*_c_ is the power required to climb and *P*_s_ is the power gained from sinking.

In an attempt to answer how much shifting altitude adds to the energetic cost of flight for birds, we made use of a simple model presented by Norevik et al. (2021) (Fig. 2). The model was originally used to estimate the energetic cost of single exploratory shifts in flight altitude by European nightjars. After a minor alteration, we expanded its use to estimate the added energetic cost of all altitude shifts for complete flights. In our model, the energy required for the flight including all shifts in flight altitude is compared with the energetic cost of the flight if it were level.

To do so, we calculated the cumulative altitude climbed (*z*), the median climb rate (*V_z_*_c_) and the median sink rate (*V_z_*_s_) of all flights. Knowing the vertical distance the bird has climbed and then sunk, and at what vertical speed, we can calculate the time spent climbing (*t*_c_) and the time spent sinking (*t*_s_) by dividing *z* by *V_z_*_c_ and *V_z_*_s_, respectively. With this information, we can first estimate what it would have cost the bird to cruise at level flight over the same time it took to climb up to and down from altitude *z*. If we assume that the bird flies at maximum range speed (*V*_mr_), the energy required for level flight can be expressed as the power it takes to fly at maximum range speed (*P*_mr_) plus the power the bird needs to sustain its basal metabolic rate (*P*_met_) multiplied by the time the bird spends flying. To calculate the energetic cost for the whole flight, we would need to first multiply the power required for level flight by the time the bird spent climbing (*t*_c_) and then multiply the power for level flight by the time spent sinking (*t*_s_). This gives us an expression of the energetic cost if the flight performed had been horizontal (i.e. level; *E*_l_):

(1) $$E_{l}=t_{c}(P_{\mathrm{mr}}+P_{\mathrm{met}})+t_{s}(P_{\mathrm{mr}}+P_{\mathrm{met}}).$$

This is of course a hypothetical scenario as a completely level flight is not possible for a species crossing the topography of Europe and Northern Africa. Nevertheless, as a null hypothesis, it is useful. If we then wish to add a climb to this equation, we need an expression for climbing power (*P*_c_). Climbing power is the additional power it takes for a bird with a body mass *m* to overcome the acceleration due to gravity ***g*** at a vertical speed of *V_z_*_c_ (Hedenström and Alerstam, 1992). A climbing bird would also have to overcome induced drag, the component of drag which arises from the difference in angle between the local airflow of the wing, created by downwash, and the free stream airflow. To account for the extra power required to overcome induced drag, we add an induced drag factor *k* to the equation. Climbing power *P*_c_ can thus be expressed as follows:

(2) $$P_{c}=mgV_{zc}k.$$

After adding a climb to the equation, we need to add a sink phase back from the total height climbed for the equation to be complete. In active sinking flight, we assume that the bird converts the potential energy gained from climbing into aerodynamic work to overcome the induced drag and therefore an induced drag factor does not apply to sinking. The power gain associated with sinking flight, sinking power (*P*_s_), can be written as:

(3) $$P_{s}=mgV_{zs}.$$

Note here that sinking speed *V_z_*_s_ is always negative; hence, sinking power *P*_s_ is subtracted as a power gain in the equation. If climbing power *P*_c_ and sinking power *P*_s_ are added to the equation for level flight, we get an expression for the energetic requirements of performing the flight, including the total shifts in altitude (*E*_c_):

(4) $$E_{c}=t_{c}(P_{\mathrm{mr}}+P_{\mathrm{met}}+P_{c})+t_{s}(P_{\mathrm{mr}}+P_{\mathrm{met}}-P_{s}).$$

We divide the cost of the flight including all altitude shifts (*E*_c_) by the theoretical cost of level flight (*E*_l_) to get the total added cost of shifting flight altitude in proportion to level flight (Fig. 2). This way of calculating the cost accounts for any differences in distance between flights. The added cost of shifting flight altitude compared with horizontal flight is thus:

(5) $$\frac{E_{c}}{E_{l}}=\frac{t_{c}(P_{\mathrm{mr}}+P_{\mathrm{met}}+P_{c})+t_{s}(P_{\mathrm{mr}}+P_{\mathrm{met}}-P_{s})}{t_{c}(P_{\mathrm{mr}}+P_{\mathrm{met}})+t_{s}(P_{\mathrm{mr}}+P_{\mathrm{met}})}.$$

### A hypothetical great snipe flight

We make several assumptions in our model, the first of which is that great snipes fly at *V*_mr_, the speed that takes a bird the furthest on a given amount of fuel. This is at the low end of how fast great snipes would fly in their long non-stop flights, where they reach ground speeds of 15–27 m s^−1^ (Klaassen et al., 2011). The estimated *V*_mr_ (15.8 m s^−1^) does however match ground speeds observed in great snipes carrying GPS tags well. To facilitate our calculations, we decided to keep this assumption, as we aim to provide a rough estimate of the cost of shifting flight altitude rather than providing exact numbers. We calculated maximum range speed, *V*_mr_, and the power needed to fly at this speed, *P*_mr_, for our hypothetical great snipe using the function *computeFlightPerformance* in the R-package *afpt* (Klein Heerenbrink et al., 2015; https://CRAN.R-project.org/package=afpt). The package *afpt* calculates flight performance characteristics from biometrical data based on a flight mechanical model of a flying bird tested in a wind tunnel (Klein Heerenbrink et al., 2015; https://CRAN.R-project.org/package=afpt). The parameters we used in our model are thus modelled rather than measured. Our hypothetical great snipe has a standard wingspan of 0.482 m and a wing area of 0.0337 m^2^, as given by *afpt* (Klein Heerenbrink et al., 2015; https://CRAN.R-project.org/package=afpt). It is about to undertake a long non-stop flight and therefore has a high body mass (Table 2). To avoid underestimating the cost of shifting flight altitude, we used the highest recorded body mass for a great snipe in Scandinavia, 260 g (Cramp and Simmons, 1983). These parameters give a maximum range speed of 15.8 m s^−1^ and a maximum range power of 4.68 W. The basic metabolic power *P*_met_ of a non-passerine can be estimated with the following formula (Pennycuick, 1975):

(6) $$P_{\mathrm{met}}=3.79\times m^{0.723}.$$

**Table 2.** Parameters and their values used to calculate the cost of changes in flight altitude

Parameter	Description (unit)	Value
*m*	Body mass (kg)	0.26
Wingspan	Average great snipe wingspan (m)	0.48
Wing area	Average great snipe wing area (m^2^)	0.034
*V*_mr_	Maximum range flight speed (m s^−1^)	15.8
*P*_mr_	Maximum range power (W)	4.68
*P*_met_	Basal metabolic rate power (W)	0.33
***g***	Gravitational constant (m s^−2^)	9.81
*k*	Induced drag factor	1.2

With a body mass (*m*) of 0.260 kg, our hypothetical great snipe would need a power of 1.43 W to sustain its basal metabolic rate. We assume the gravitational constant to be constant and set at sea level (9.81 m s^−2^) which should only have minor effects on our results. All the parameters for our hypothetical great snipe can be found in Table 2. The model assumes that great snipes use flapping flight throughout the entire flight. This is supported by high activity levels recorded by the accelerometers in the loggers.

There have been few attempts at experimentally determining the value of the induced drag factor *k* in flying birds. Suggestions range from 1.04 (Spedding, 1987) to above 1.4 (Klein Heerenbrink et al., 2015) and here we decided to follow Pennycuick's (1975) recommendation of 1.2. However, the amount of induced drag might influence the cost of shifting flight altitude, so we calculated costs for a value of *k* of 1.04, 1.1, 1.2 and 1.43 and compare the results in Table S3.

Using these parameter values, we can calculate the added cost of shifting flight altitude for a hypothetical flight by a great snipe. Let us say that during this flight, the snipe climbed a total of 5000 vertical metres (*z*=5000 m) at a climb rate of 0.5 m s^−1^ (*V_z_*_c_=0.5 m s^−1^). For simplicity, it landed at the same height above sea level as it took off and had an average sink rate of 0.5 m s^−1^ (*V*_*z*s_=−0.5 m s^−1^). The time spent climbing is then 10,000 s (*t*_c_=5000/0.5) and the time spent sinking is also 10,000 s (*t*_s_=5000/0.5). Once we have the time spent climbing and the time spent sinking, we can calculate the energy requirements for a level flight (*E*_l_) of the same duration using Eqn 1, with the values for *P*_mr_ and *P*_met_ from Table 2:

(7) $$E_{l}=10,000(4.68+0.33)+10,000(4.68+0.33).$$

It takes the snipe 100,200 J to perform this level flight. We need to know the climbing power and sinking power to add an altitude shift to the equation. The climbing power for our hypothetical great snipe depends on its body mass (*m*=0.26 kg), the gravitational constant, where we assume ***g***=9.81, its climb rate (*V_z_*_c_=0.5 m s^−1^) and the induced drag factor, which is set to *k*=1.2. Using Eqn 2, this gives us the following climbing power:

(8) $$P_{c}=0.26\times 9.81\times 0.5\times 1.2.$$

Put in words, the great snipe needs an additional power of 1.53 W to climb at 0.5 m s^−1^. We can calculate the gain in power from sinking in a similar way (Eqn 3), assuming the same value for body mass and the gravitational constant, with a sink rate of 0.5 m s^−1^:

(9) $$P_{s}=0.26\times 9.81\times 0.5.$$

The power gain from sinking is then 1.28 W. We can now calculate the energy required for the flight including the altitude shift when we know the climbing power and sinking power gain. The energy for a flight with an altitude shift (*E*_c_) can be expressed with the equation for level fight with the addition of climbing and sinking power:

(10) $$\begin{array}{r} E_{c}=10,000(4.68+0.33+1.53)+10,000(4.68+0.33-1.28). \end{array}$$

Our hypothetical snipe will use up 102,400 J when performing this flight with an altitude shift included. But calculating the exact energy requirements for a flight is not what we aimed for with this study. The energy a bird uses in a flight will largely depend on the environmental conditions in which the flight is performed, such as the temperature and wind conditions. As we do not have positions for our birds, we cannot make accurate energy calculations in a sensible way. Instead, we compared the ratio between the energy required for a flight including attitude shifts and that required for the same flight if it were level (see Eqn 5):

(11) $$\frac{E_{c}}{E_{l}}=\frac{10,000(4.68+0.33+1.53)+10,000(4.68+0.33-1.28)}{10,000(4.68+0.33)+10,000(4.68+0.33)}.$$

The snipe in our example adds a percentual energy cost of 2.49% by shifting altitude at a climb rate of 0.5 m s^−1^ while sinking at 0.5 m s^−1^. This percentual added cost is not expected to change to any noteworthy extent with flight conditions.

## RESULTS

### Flight altitude shifts – how often and how large?

Great snipes had a mean (±s.d.) vertical tortuosity of 0.48±0.11 for the full dataset with a 1 h sampling interval. This means that on average they switched between climbing and sinking or vice versa once every other hour. They made altitude shifts just as often in all their marathon flights (*F*_2,2_=2.58, *P*=0.08) and they did not shift altitude more often during the night than in the day, or any time in between (*F*_2,2_=0.05, *P*=0.95). For the 5 min sampling interval, the mean (±s.d.) altitude tortuosity of a flight was 0.30±0.05, corresponding to a shift every 17th minute.

**Fig. 3.**
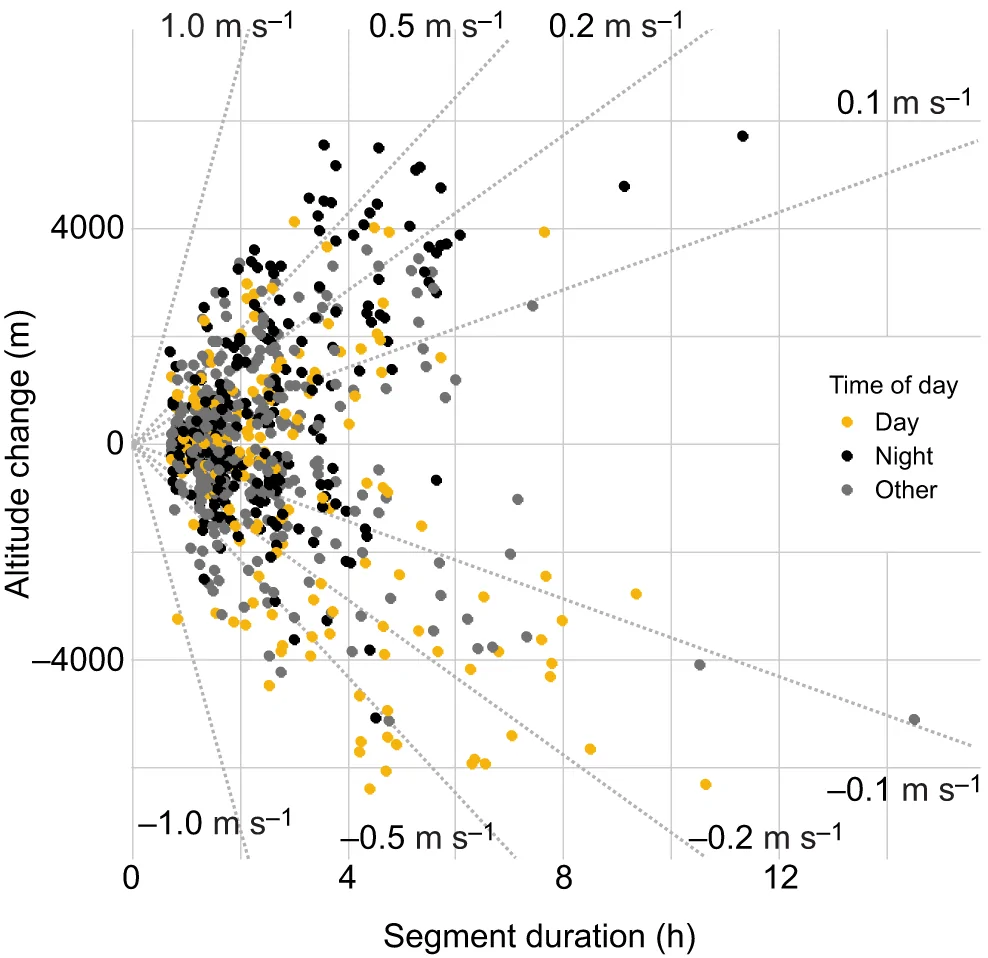
**The 1** **h climb and sink rates plotted against segment duration.** Dashed grey lines indicate climb and sink rates equal to 0.1, 0.2, 0.5 and 1.0 m s^−1^ and the colour of the datapoints is determined by the time of day at which the segment was started. Yellow points are the daytime segments (08:00–14:00 h UTC), black points mark night-time segments (20:00–02:00 h UTC) and grey points show any time in between, here called ‘other’.

The median altitude shift of a great snipe segment was 723 m (IQR=±245 m). The birds did not perform larger continuous shifts in flight altitude in the night than in the day or any other time of day (Fig. 3; *F*_2,2_=0.08, *P*=0.93), nor did the flight altitude shifts differ between the three marathon flight types (*F*_2,2_=2.00, *P*=0.14).

### Flight altitude shifts – how fast?

**Fig. 4.**
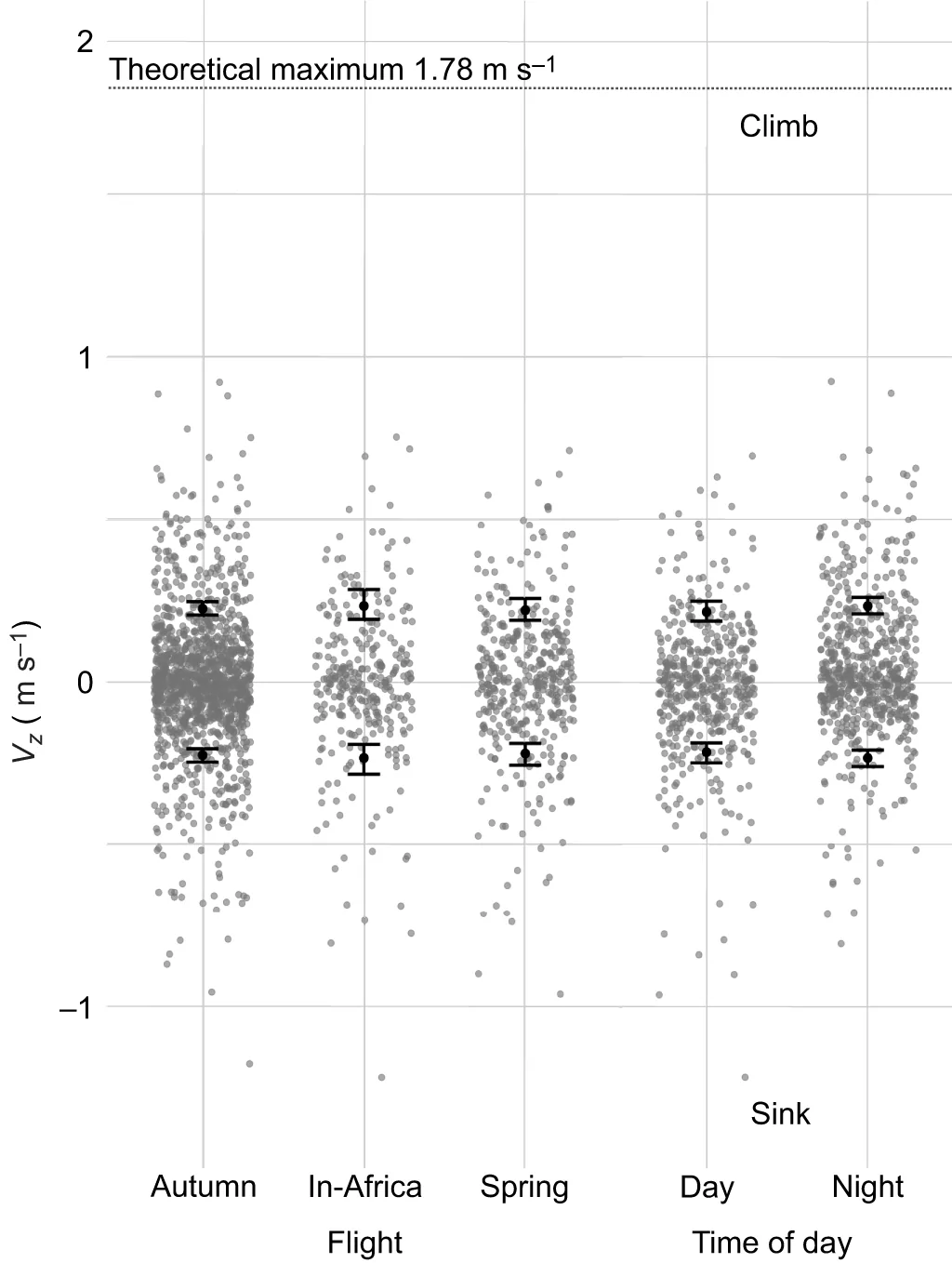
**Climb and sink rates for the three marathon flights and during the day and night.** Black points are back-transformed estimated marginal means from the log-linear model described in Materials and Methods, and error bars show their 95% confidence intervals. The theoretical maximum climb rate for great snipes flying at *V*_mr_ (Klein Heerenbrink et al., 2015) is shown with a dashed grey line.

Great snipes climbed at a median rate of 0.21 m s^−1^ (IQR=0.19), which equals a 756 m gain in elevation per hour (Table S2). The fastest climb performed by a snipe in our dataset with a 1 h sampling interval was 0.92 m s^−1^ (Figs 3 and 4). In this climb, the bird gained 3327 m of elevation in 1 h, as part of a 4 h long continuous climb from 927 to 6472 m.a.s.l. during its autumn marathon flight. The snipes equipped with 5 min loggers climbed at a median rate of 0.28 m s^−1^ (IQR=0.29; Table S2), while the fastest climb rate recorded in the 5 min subset was 3.45 m s^−1^ (Fig. S3).

When great snipes sank, they did so at a median rate of 0.22 m s^−1^, which corresponds to a 792 m drop in altitude per hour (Table S3). The steepest sink was 1.22 m s^−1^, where the snipe lost 4383 m of elevation in 1 h at the end of a 21 h intra-African flight. For the snipes tracked with a 5 min sampling interval, the median sink rate was 0.27 m s^−1^ and the fastest drop in altitude was a sink rate of 6.48 m s^−1^ (Table S3).

We found similar climb rates for all three marathon flights (Fig. 4; *F*_2,1_=0.09, *P*=0.91). Climbs at night were not faster than climbs in the day (Figs 3 and 4: estimated difference=−0.08, *P*=0.34). For sinking flight, great snipes showed similar rates in the night and day (*F*_2,1_=0.90, *P*=0.35). There were no differences in sink rate between the three marathon flights (Fig. 4: *F*_2,1_=3.01, *P*=0.054).

**Fig. 5.**
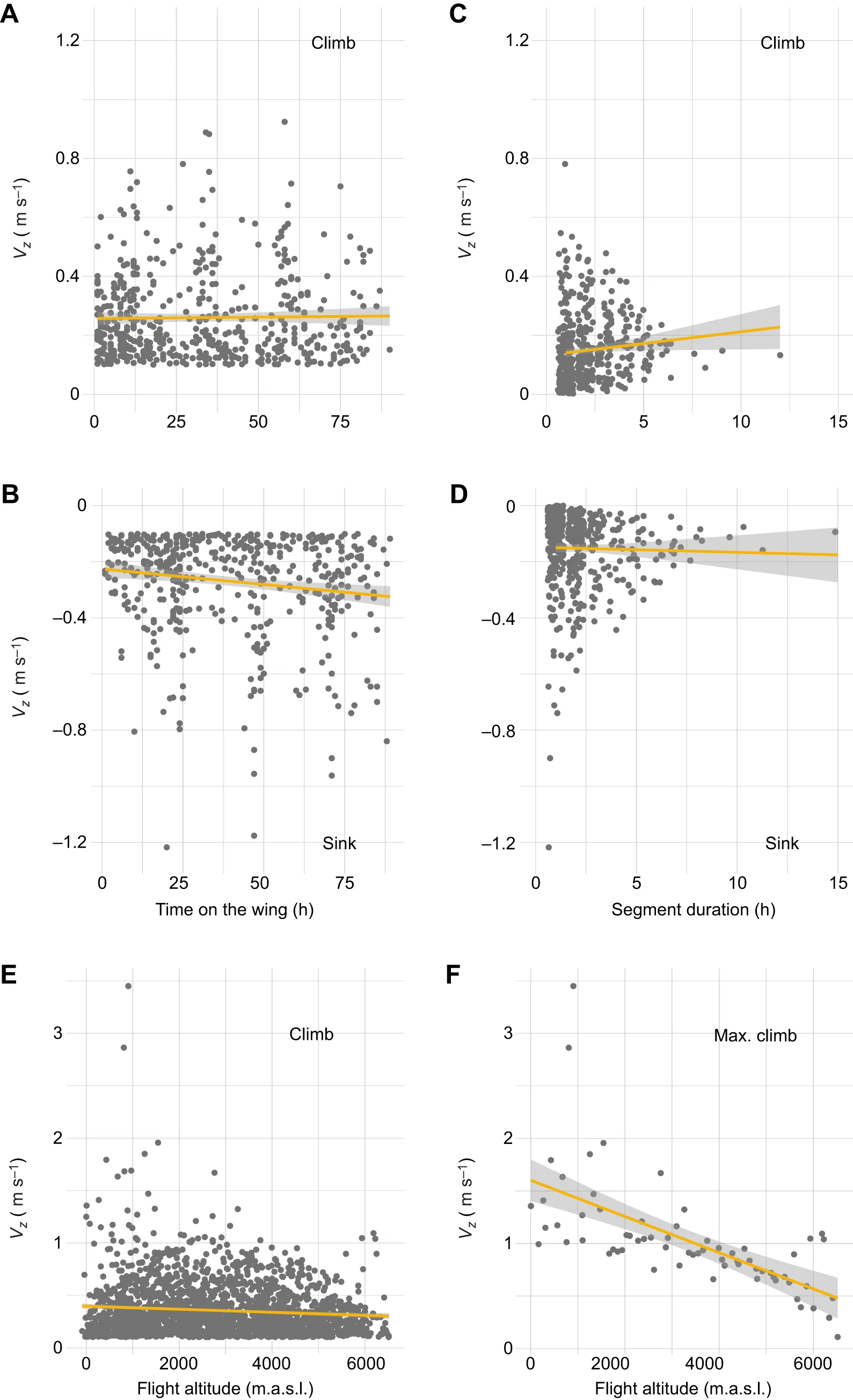
**Climb and sink rates in relation to time on the wing, duration and altitude.** (A,B) Climb and sink rates plotted against the time the birds had been airborne. (C,D) Mean climb and sink rates for each continuous climb or sink segment plotted against the length of the segment. (E) Climb rate plotted against flight altitude. For A–E, a linear model was fitted to the data in each plot for visual purposes and is shown in yellow with standard errors shaded in grey. (F) Maximum observed climb rate for each 100 m of altitude. The linear model regression line from the test is shown in yellow with corresponding standard errors shaded in grey. All data included in the figure have a sampling interval of 1 h.

Great snipes did not climb faster at the end of their marathon flights than at the beginning (Fig. 5A), as we found no effect of total time on the wing on their climb rates (*F*_1,507_=0.003, *P*=0.956). They did however sink at significantly faster rates the longer they had been flying (Fig. 5B; *F*_1,402_=14.18, *P*=0.002).

The longer the duration of a climb segment, the faster on average the great snipes climbed (1 h resolution: *F*_1,456_=23.24, *P*<0.001), a pattern consistent in both the 1 h (Fig. 5C) and the 5 min sampling subsets (5 min subset: *F*_1,491_=53.46, *P*<0.001). Similarly, great snipes sank at faster rates in longer sink segments than in short segments (Fig. 5D), both when analysing the data for 1 h resolution and in the 5 min subset (1 h resolution: *F*_1,443_=10.96, *P*=0.001; 5 min subset: *F*_1,490_=75.70, *P*<0.001).

We found that great snipes climbed at lower rates the higher they flew. This negative relationship was statistically significant both when analysing all climb rates (*F*_1,1717_=8.96, *P*<0.003; Fig. 5E) and for the model of maximum observed climb rate per 100 m of altitude (*F*_1,64_=62.1, *P*<0.001; Fig. 5F). Including all climb rates, great snipes climbed 0.016 m s^−1^ slower per kilometre of altitude. When looking at maximum observed climb rates, great snipes climbed 0.17 m s^−1^ slower per kilometre of elevation gained.

The maximum climb rates from all flights in the 5 min subset did not differ from the theoretical maximum climb rate (median=1.20 m s^−1^, *U*=13, *P*=0.16). However, the highest maximum climb rate recorded in our 5 min sampling interval dataset was almost twofold the theoretical maximum rate (3.45 m s^−1^). When we treated this datapoint as an outlier and excluded it from the test, the maximum climb rate great snipes performed was significantly lower than the theoretical maximum climb rate (median=1.15 m s^−1^, *U*=3, *P*=0.02). The 5 min loggers recorded higher median climb rates (1 h median=0.21 m s^−1^, 5 min median=0.28 m s^−1^, *W*=293,170, *P*<0.001) and sink rates (1 h median=0.21 m s^−1^, 5 min median=0.27 m s^−1^, *W*=279,142, *P*<0.001) than the 1 h loggers.

Throughout the analysis, differences between individuals never explained more than 5% of the variation.

### Flight altitude shifts – how costly?

We found that by shifting flight altitude, a great snipe increased the energetic cost of its migratory flight by on average 0.65±0.13% (±s.d., range 0.42–1.03%). Both the highest and lowest values occurred during in-Africa flights. The highest assumed value for the induced drag factor *k* gave the highest added costs of shifting flight altitude (Table S3). Using a *k* of 1.43, the mean added cost for all flights was 2.18%.

## DISCUSSION

We explored the climbing and sinking performance of great snipes, a species with particularly large altitude shifts during long flights, to quantify their behaviour and estimate the energy cost of their climbs and sinks.

### Flight altitude shifts – how often and how large?

Our results show that great snipes shifted flight altitude at comparable rates and scale during the three different marathon flights. Similarly, they did not shift altitude more often, or at a larger scale, at any particular time of day (day, night, other). This should however not be interpreted as an argument against the overall diel cycle in flight altitude in the species (Lindström et al., 2021; Sjöberg et al., 2023). That we did not find a difference in how frequent and how large the altitude shifts that great snipes make is explained by the broad uncertainties in our classification of time periods. The time period group ‘other’ included not only the immediate periods of dusk and dawn, when great snipes most frequently perform large shifts in flight altitude, but also parts of true day and night periods. Thus, the group ‘other’ also includes ‘missclassified’ periods of time when we do not expect any large shifts in flight altitude to take place, which can lower the median altitude change for this group. Outr results for the group ‘other’ should therefore be interpreted carefully. What our results do show is that great snipes make large and frequent altitude shifts even when they have reached their daytime or night-time flight altitude.

Hypotheses for why they would make large shifts in flight altitude between day and night in a diel cycle are discussed in detail elsewhere (Lindström et al., 2021; Sjöberg et al., 2021, 2023). However, the shifts in altitude within the day or night probably have other ecological drivers. The reason behind these shifts in flight altitude within the day or night is unclear. It is reasonable to expect that they are related to variation in weather conditions at different altitudes. Migratory birds have been shown to aggregate at altitudes with more favourable wind conditions in radar studies (Gauthreaux, 1991). Additionally, Senner et al. (2018) found that black-tailed godwits, *Limosa limosa*, track a combination of wind and temperature conditions, arguing that flight altitude decisions are probably influenced by an interaction of multiple weather conditions. It has also been suggested that during the night, nightjars make ‘exploratory shifts’ in flight altitude, perhaps to sample wind conditions aloft (Norevik et al., 2021).

The fact that the great snipes did not climb slower in the daytime than at night indicates that they were not constrained by solar radiation during their climbs. We see two possible, not mutually exclusive, explanations for this. The first is that the large difference in flight altitude, and thus in temperature, between day and night might already counteract this effect. The second is that they may adjust their climb and sink rates to minimize the heat produced in these shifts. Quantifying how often and at what scale other taxa make within-daytime and -night-time altitude shifts is a good place to start for understanding why birds shift flight altitude. What is clear, however, is that great snipes rarely maintain a constant flight altitude in potentially suitable wind conditions, as has been assumed for migratory birds in general (Hedenström and Alerstam, 1994).

### Flight altitude shifts – how fast?

One way to reduce the cost of constant changes in flight altitude could be to perform these climbs and sinks at low rates. Great snipes seem to shift altitude relatively slowly, apart from the occasional high maximum climb and sink rates. Jenni-Eiermann et al. (2024) found low sink rates for nocturnal songbird migrants over Europe. They argued that birds reduce the mechanical cost of flight by 11–30% by sinking slowly in the 1.5 h before their final descent and found support for this lower cost in the metabolites (as indicators of fuel use) of birds caught mid-flight over an Alpine pass. Overall, low vertical speeds would thus lower the energetic cost associated with shifting flight altitude, meaning that climbing and sinking at low rates could indeed be an adaptive behaviour to save energy and avoid overheating, or both.

The snipes did not climb faster the longer they had been flying, as we were expecting from a flight mechanics perspective. A lower body mass due to consumed fuel stores should leave the snipes with a larger power margin to climb, the longer into the flight they are (Hedenström and Alerstam, 1992). Our results give us no reason to assume that snipes take advantage of this lower cost of climbing at the end of flights to get to their desired altitude quicker. We suggest two possible explanations for these uniform climb rates throughout the flight. The benefit of a lower body mass is counteracted by a loss of power available in the flight muscles. Birds consume not only fat during their migratory flights but also protein (including flight muscle mass), potentially leading to a loss of flight muscle performance (Battley et al., 2000). However, it seems as though great snipes perform most of their shifts in altitude at a low rate, well below their maximum capacity. The general lack of near-maximal climbs, both in the beginning and at the end of flights, points toward this being a behavioural adjustment, rather than an effect of impaired physiological performance. This controlled manner of gaining and losing elevation seems to be uniform across all marathon flights in the species. The somewhat higher sink rates at the end of some flights could be said to be an exception to this, even though the majority were still below −0.4 m s^−1^ (Fig. 5B). Fast sink segments were often performed at the very end of marathon flights, including the maximum sink rate of 6.48 m s^−1^. It has been shown for several songbird species that birds can stoop down quickly at the end of migratory flights (Hedenström and Liechti, 2001; Jenni-Eiermann et al., 2024). Even at night, during which we observed the fastest sinks in the great snipes, birds seem to be able to locate suitable stopover habitat from aloft, probably using visual cues (Jenni, 1996). These fast descents are probably the reason why we found that sink rates increase with time on the wing in great snipes. But compared with the maximum sink rates of on average >30 m s^−1^ found in songbirds (Hedenström and Liechti, 2001), more equal to stoops, even the highest great snipe sink rates would still be considered low.

Our results suggest that great snipes climb slower the higher they fly, when considering both their average, submaximal behaviour and their fastest climbs. The negative slope of −0.17 m s^−1^ per kilometre for maximum observed climb rates falls well in line with the hypothesis that a lower air density would leave a smaller power margin to climb and thereby limit a bird's climbing performance (Pennycuick, 1975). Our estimated slope corresponds to an 11% decrease in maximum observed climb rate per kilometre of altitude gain, which is a steeper negative relationship than the expected 5%. It should be noted that our prediction of a 5% decrease is based on an ideal bird in standard atmospheric conditions. A slight mismatch in slope between ideal and real-life conditions is only to be expected.

When it comes to the relationship between all climb rates and flight altitude, it is important to bear in mind that the climb rates we found are well below the theoretical maximum of the species. In this test, we are therefore not looking at limits to the exercise performance of great snipes, but rather at their behaviour. The negative relationship we found is statistically significant but the biological relevance of a 16 m difference in elevation gain for a 1 h long climb is questionable.

Contrary to our prediction, great snipes did not climb faster in the night than in the day, despite a 2000 m difference in mean flight altitude (Table S1). Lower oxygen concentrations, lower air pressure and colder ambient temperatures at high altitudes do not seem to affect the rates at which great snipes climb. Though we know little about avian flight at high altitude, Ivy et al. (2025) recently found that yellow-rumped warblers, *Setophaga coronata*, in a wind tunnel could fly at 75% of their maximum simulated flight altitude (as determined under experimental conditions) without showing signs of a hypoxic response. This suggests that flying at altitudes below a species' maximum altitude does not represent a respiratory challenge.

As climbing flight is more demanding than level flight, it should produce more heat. The lower ambient temperatures at the snipe's daytime flight altitudes might counteract the combined heating effect of exercise and solar radiation, allowing the snipes to climb at similar rates in the day to those at night. Our results show that for most of the flight, the snipes were climbing well below their theoretical maximum climb rate (Klein Heerenbrink et al., 2015). It is thus unlikely that any differences we found here reflect constraints to their exercise performance. Not even in their fastest climbs do great snipes reach their theoretical maximum climb rate. With a median maximum climb rate of 1.15 m s^−1^, the snipes are however faster climbers than nightjars, which had a median maximum climb rate of 0.67 m s^−1^. The fastest climbs performed by great snipes are close to the initial climb rates of similar-sized red knots departing from Iceland (1.1 m s^−1^; Hedenström and Alerstam, 1994).

It was somewhat unexpected that great snipes would climb and sink faster the longer the climb or sink segment was. The biological relevance of the still small differences in climb and sink rate with segment length (Fig. 5C,D) is however debateable. What is more remarkable is how far below the theoretical maximum the great snipes climbed and how consistent these slow vertical speeds were throughout their whole flights. The behaviour was consistent not only within individuals but also between individuals, as shown by the low amount of variation explained by individual differences (<5%). This consistency suggests that low rates of climb and sink are not accidental and that they have an adaptive benefit. If they climb or sink at low rates, it does not make a difference how often or how large the shifts in flight altitude are. This interpretation would benefit from comparable data from other taxa in order make any generalizable claims. Detailed tracking data from multisensor data loggers including barometric pressure already exist for many species (e.g. Senner et al., 2018; Briedis et al., 2020; Norevik et al., 2021; Sjöberg et al., 2021; Rime et al., 2023; Dufour et al., 2026), enabling comparisons of climb rates under various conditions.

### Flight altitude shifts – how costly?

The negligible added cost of 0.65% for shifting flight altitude may be surprising at a first glance. Our model is simple and based on many assumptions, meaning that we make no claim to have found the true energetic costs. It does however serve the purpose of giving a rough estimate of the magnitude of altitude shift costs. The costs associated with high altitude flight, especially in combination with varying ambient temperatures are still poorly understood and would benefit from being tested under experimental conditions. We further note that the model could be used to calculate cumulative climbing and sinking costs at every shift in altitude rather than summarizing flights into mean climb and sink rates. This would give added costs closer to real values, but they would most probably still be low.

Low costs associated with vertical displacements were also found in European nightjars (Norevik et al., 2021). The authors note that the lowest cost for shifting altitudes would be attained at slow climb and sink rates and, if the birds continue to fly, by flapping flight during the descent. This fits well with the climbing and sinking behaviour we report for great snipes here, with slow shifts in altitude, well below the theoretical maximum, and a sustained flapping flight while sinking (Fig. 1D). By shifting altitude at low rates and maintaining active flight while sinking, great snipes can turn the potential energy gained from climbing into kinetic energy to reduce induced drag when sinking, thus conserving most of the energy. Also, European nightjars (Norevik et al., 2021) and black-tailed godwits (Senner et al., 2018) remain in active flight while sinking, suggesting that this could be a common behaviour in migratory birds. Following our estimate of a low overall energetic cost for shifting altitude in great snipes, arguably one of the species with the highest altitude variability found to date, we suggest a low cost of shifting flight altitude for most migrants, with or without a diel altitude cycle.

### Conclusion

While we still do not fully understand why great snipes make such pronounced shifts in flight altitude, we can conclude that it is not necessarily energetically costly. The reason for this is that great snipes climb and sink at rates well below their theoretical maximum rates. Maintaining active flight while sinking at low rates could be a key behaviour in reducing the added cost of shifting flight altitude, allowing even large and repeated altitudinal shifts to be beneficial. Nevertheless, the reason for each altitude shift remains to be determined.

## Supplementary Material

10.1242/jexbio.252022_sup1 Supplementary information
