# Supplementary material for: Low cost of shifting flight altitude in migrating great snipes
Source: J Exp Biol. 2026 Aug 28;229(16):jeb252022. doi: 10.1242/jeb.252022 (PMC13571815; doi:10.1242/jeb.252022)
Supplement: Supplementary file 1 [file jexbio-229-252022-s1.pdf]

Table S1. Mean and maximum flight altitude for different times of the day. Daytime is defined as 08:00-14:00 UTC) and night as 20:00-02:00 UTC. "Other" refers to any hour in between, when great snipes are expected to perform large altitude shifts as a part of their diel altitude cycle.

Time of day	Mean altitude (masl)	SD	Maximum altitude (masl)
Day	4120.74	1698.57	8077
Night	2091.32	1280.80	6571
Other	3044.21	1723.18	7182

Table S2. Median and maximum climb and sink rates for great snipe marathon flights, excluding rates lower than 0.1 ms^{-1}. Vertical speeds obtained from the full dataset with a 1 hour sampling interval is showed above and the 5-minute subset below.

1-hour data	Median V_z (ms^{-1})	IQR	Maximum V_z (ms^{-1})
Climb	0.20	0.18	0.92
Sink	0.22	0.22	1.49
**5-minute data**			
Climb	0.28	0.29	3.45
Sink	0.27	0.25	6.48

Table S3. Mean added cost of flight altitude shifts for different values of induced drag factor k suggested in literature and the associated standard deviation (sd). The mean added cost is expressed as the percentage of added cost to a flight due to shifts in flight altitude compared to a hypothetical level flight.

k	**Mean added cost of shifting flight altitude (±sd)**	
	1 hour	*5 minutes*
1.04	0.13 (±0.03)	0.22 (±0.04)
1.1	0.32 (±0.07)	0.55 (±0.1)
1.2	0.65 (±0.13)	1.09 (±0.19)
1.4	1.3 (±0.26)	2.18 (±0.38)

Journal of Experimental Biology • Supplementary information

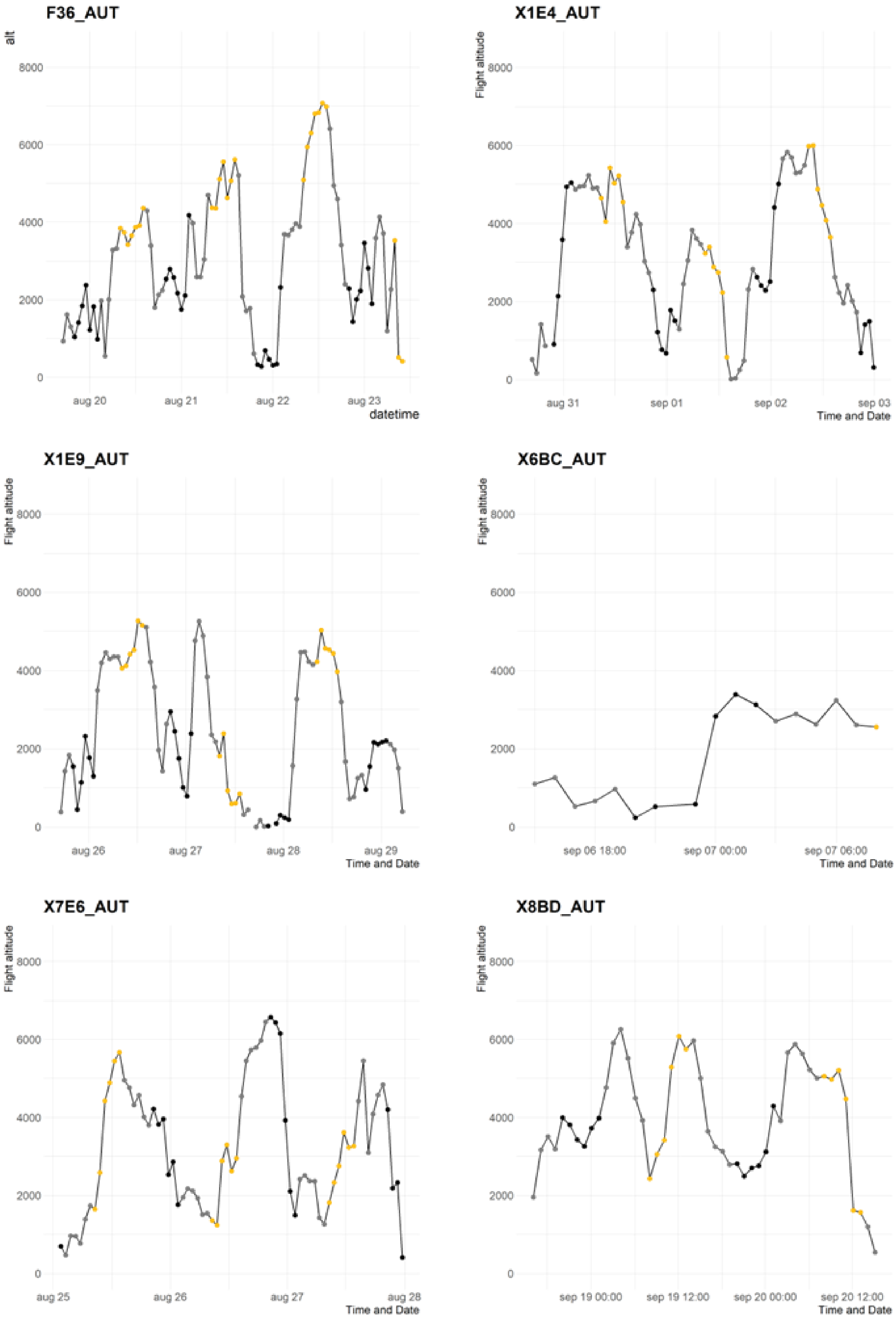

Journal of Experimental Biology • Supplementary information

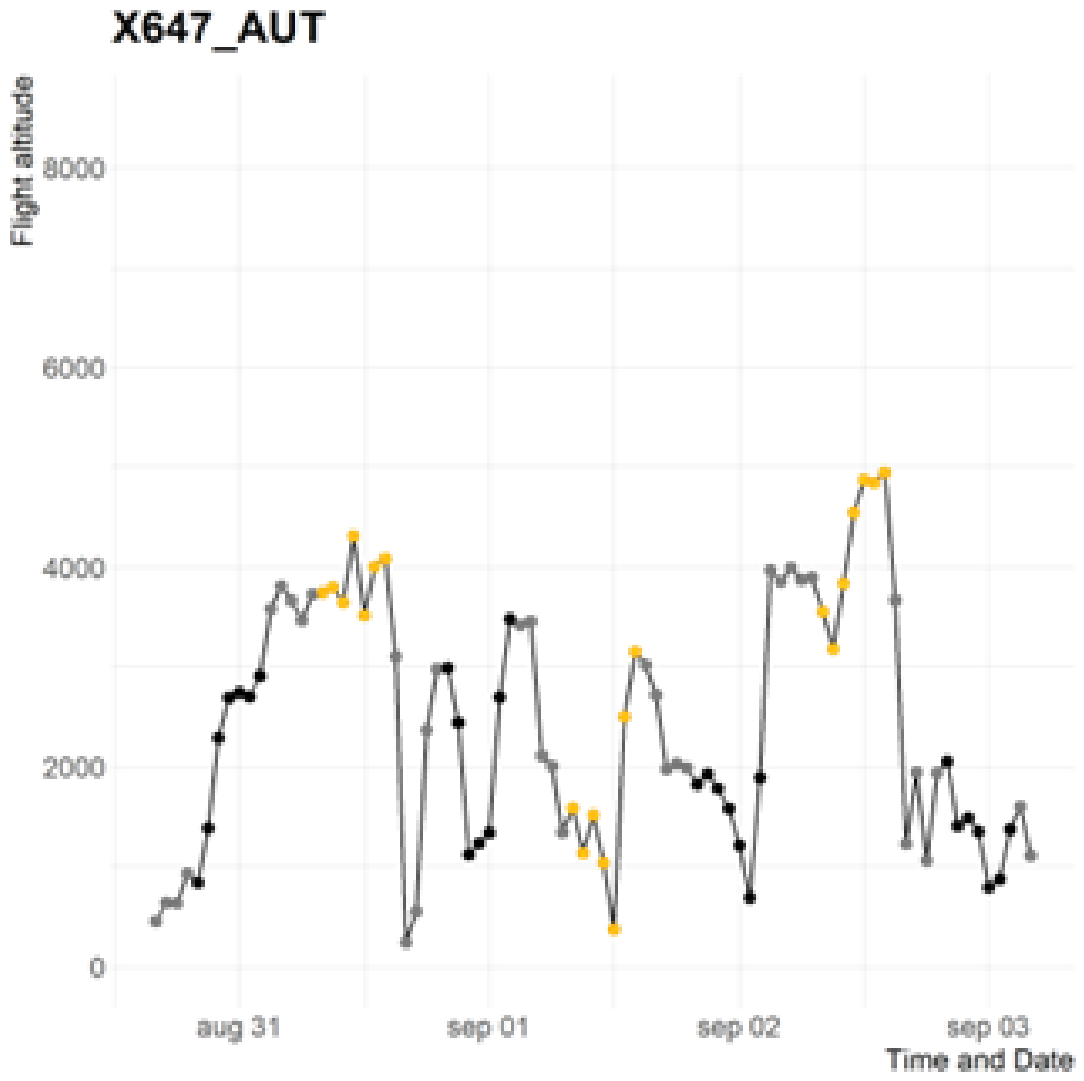

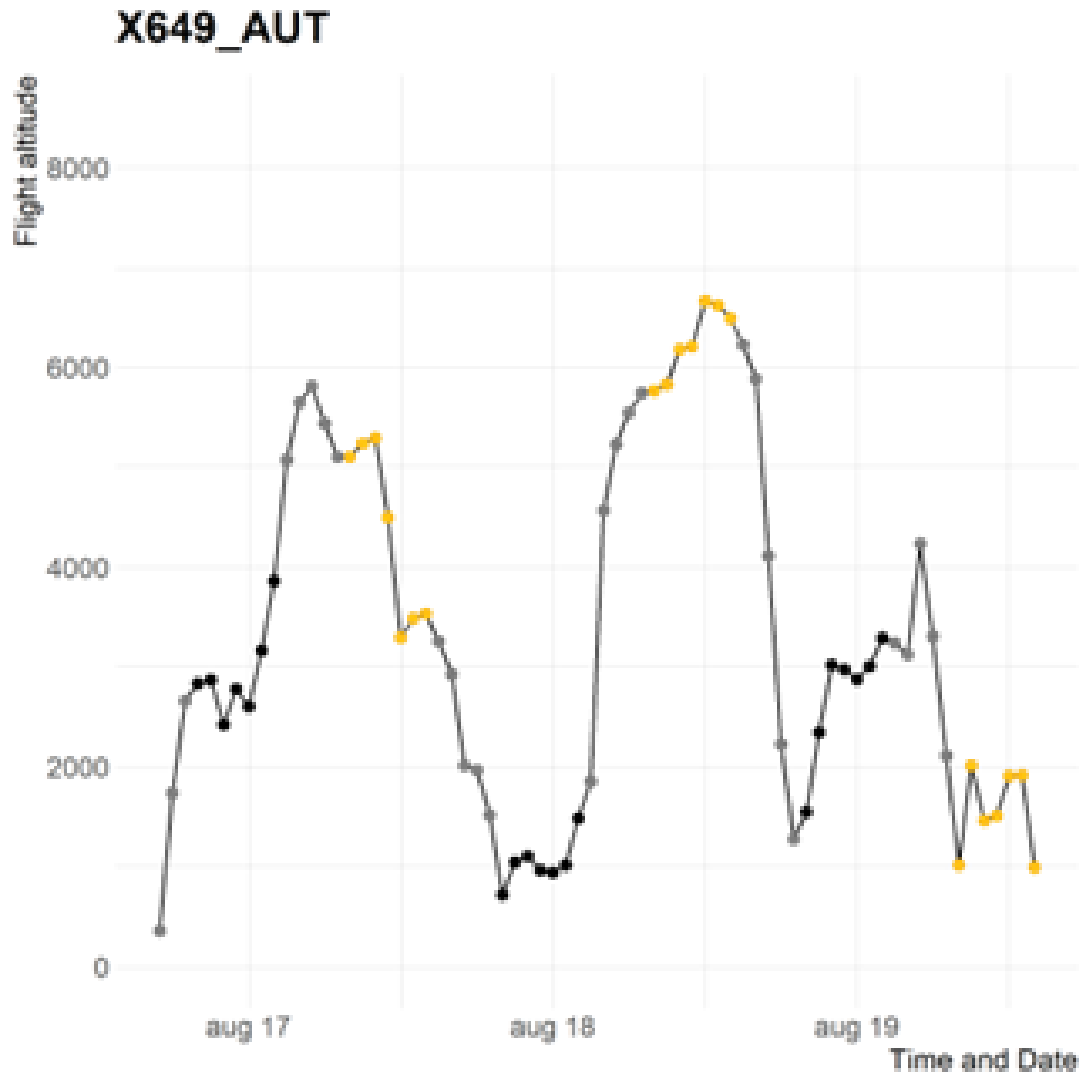

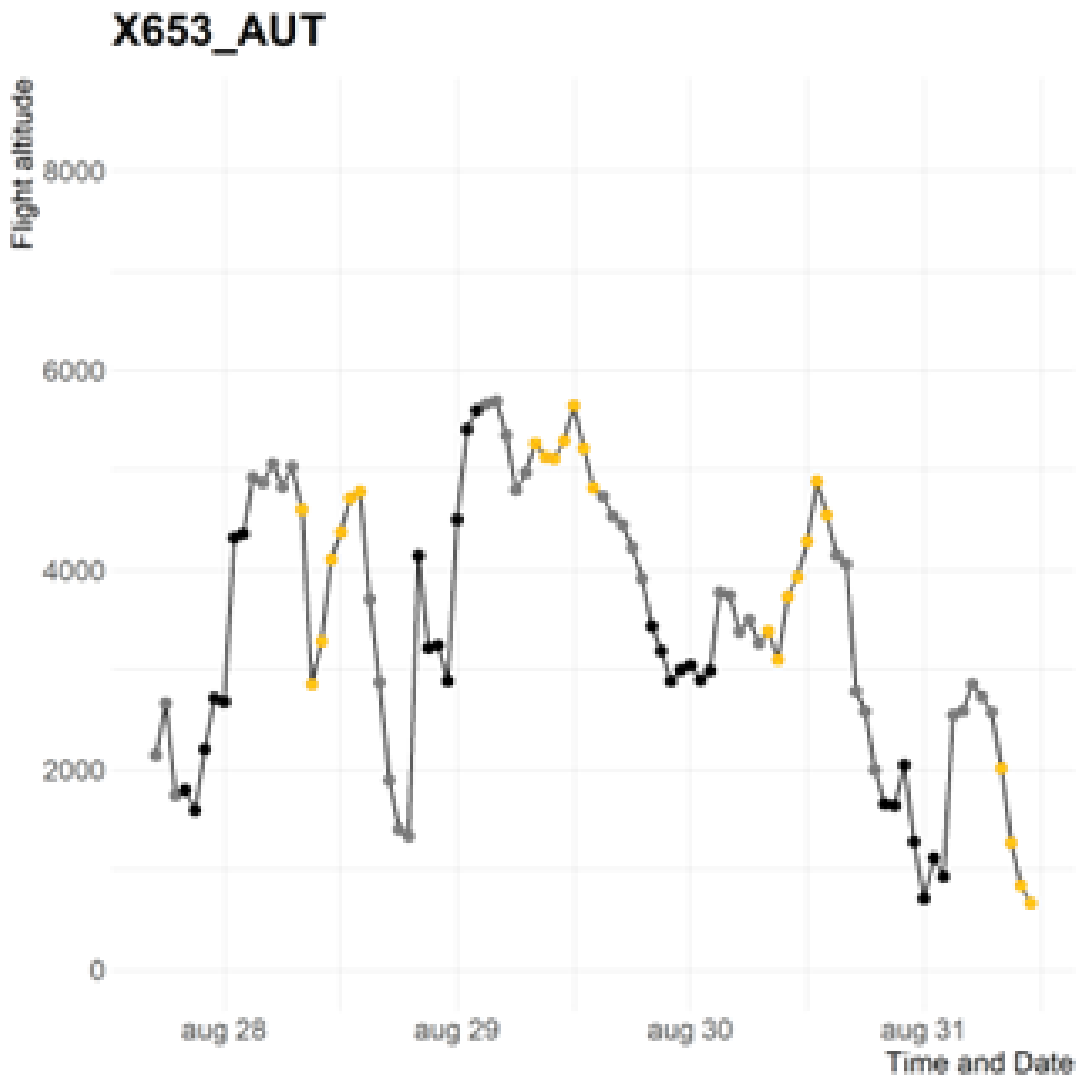

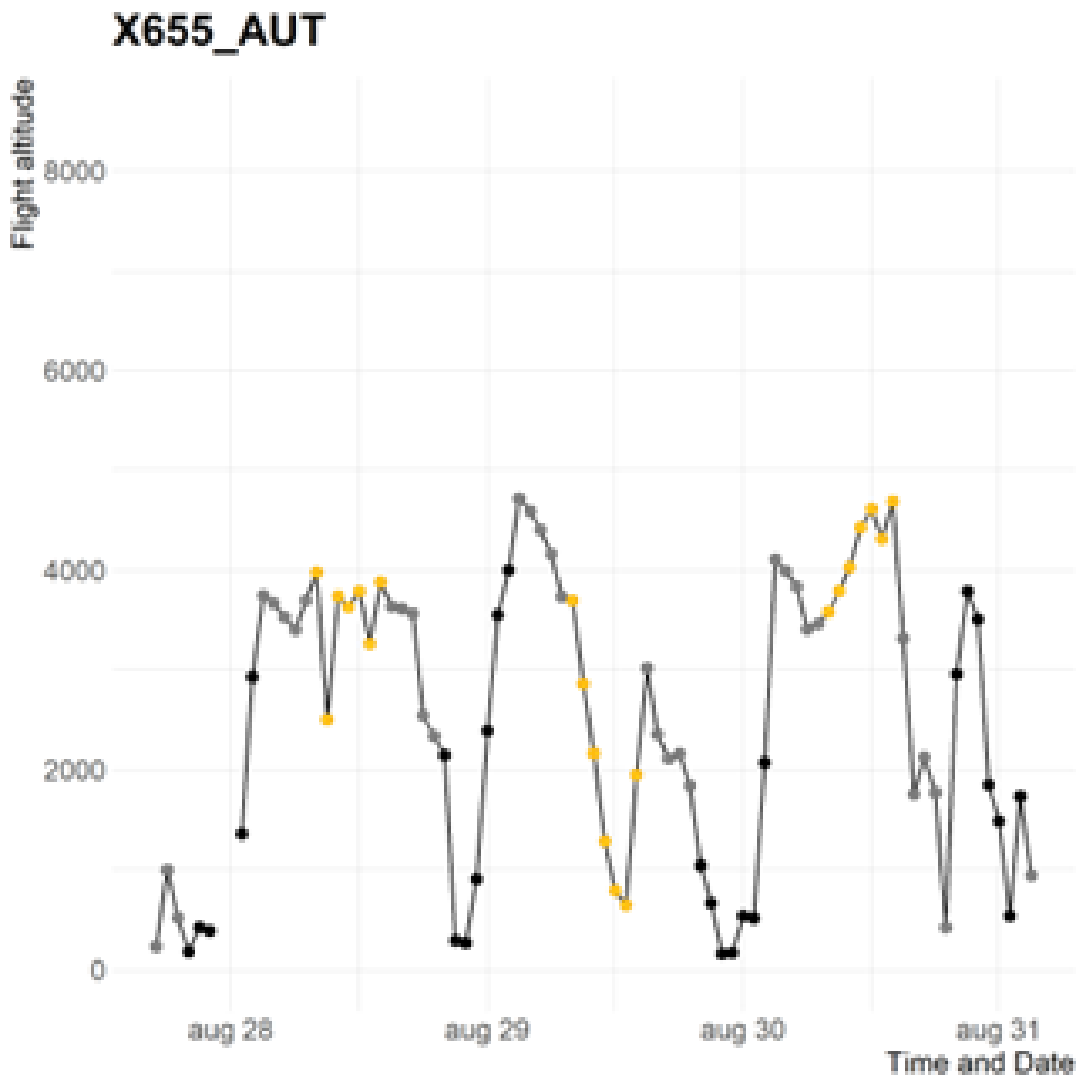

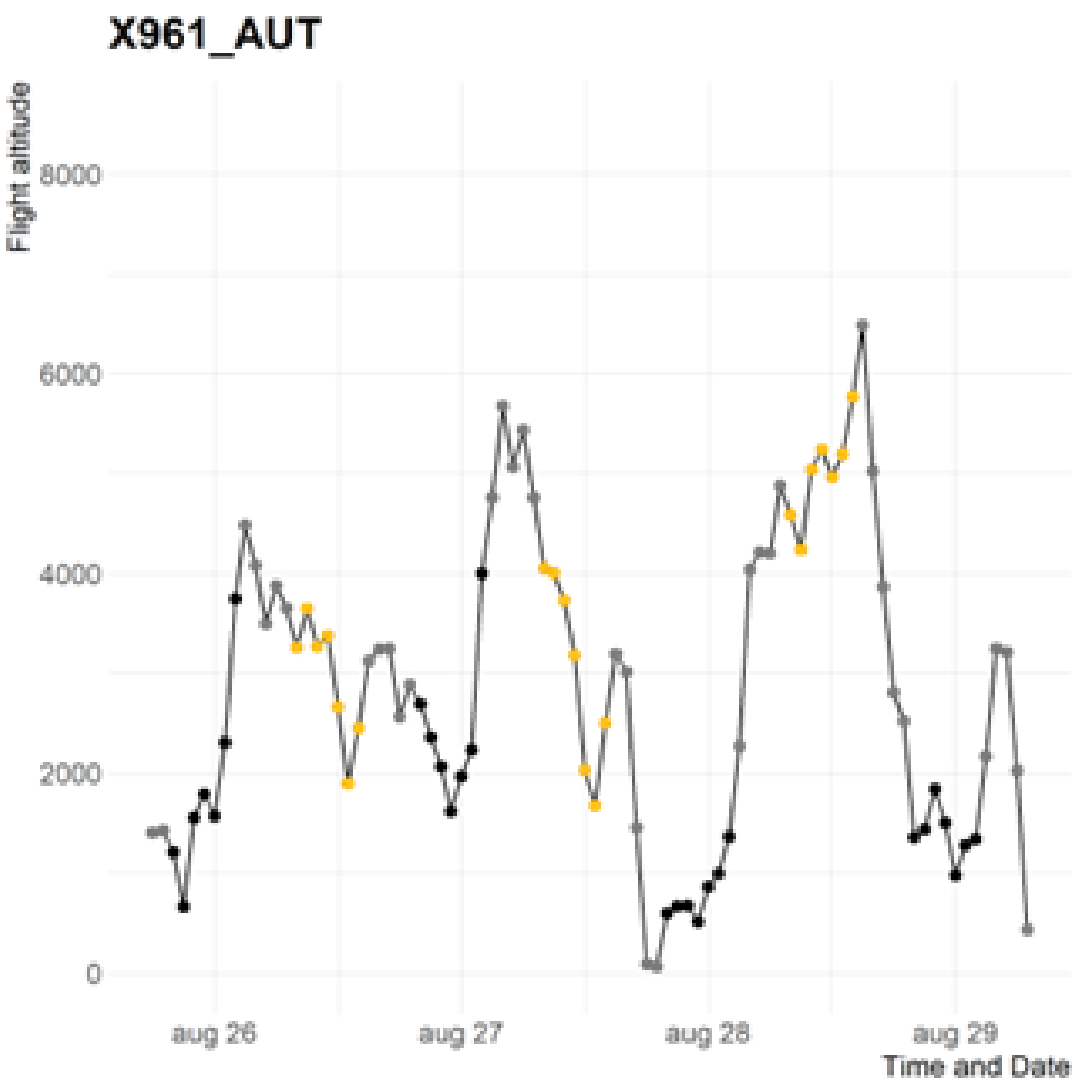

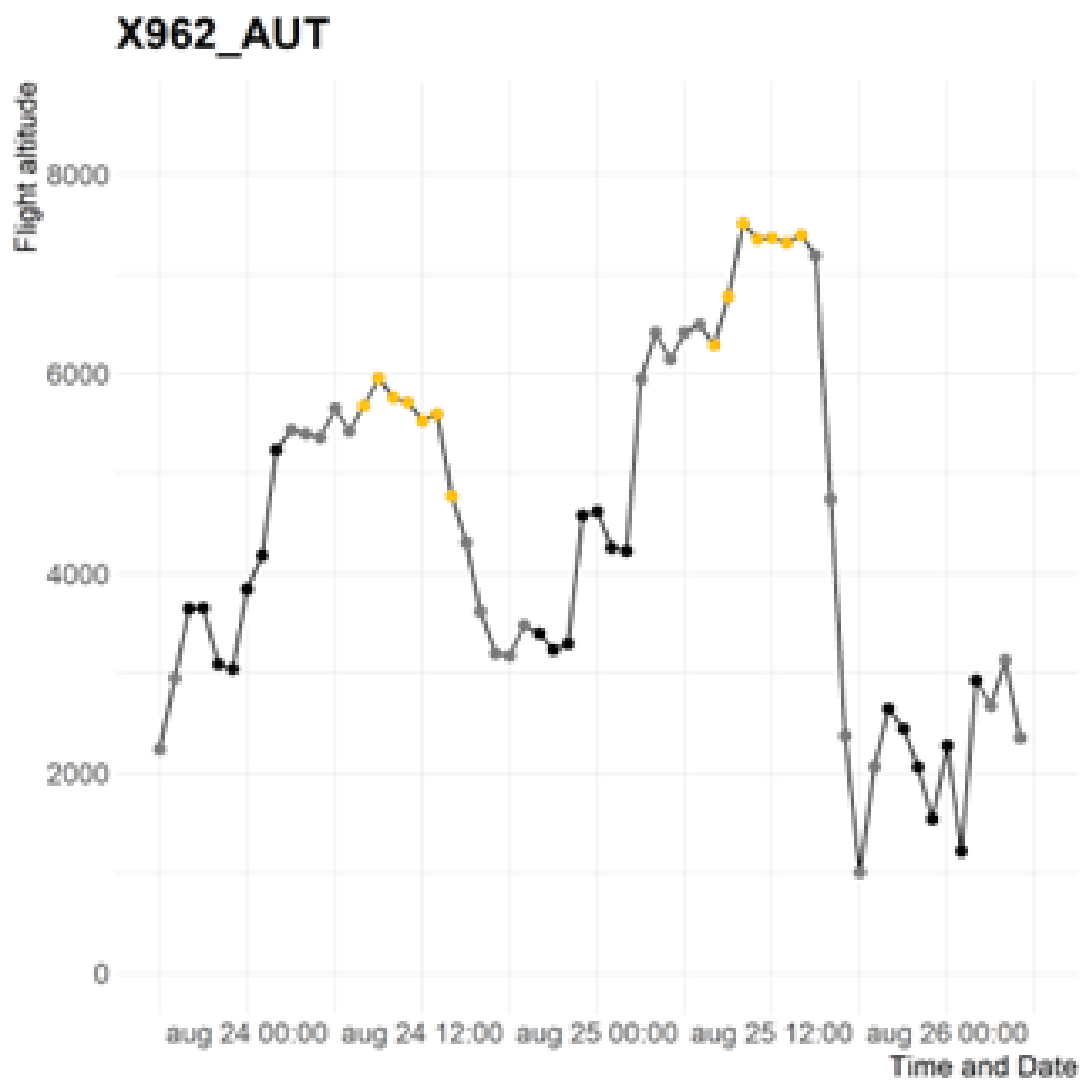

Journal of Experimental Biology • Supplementary information

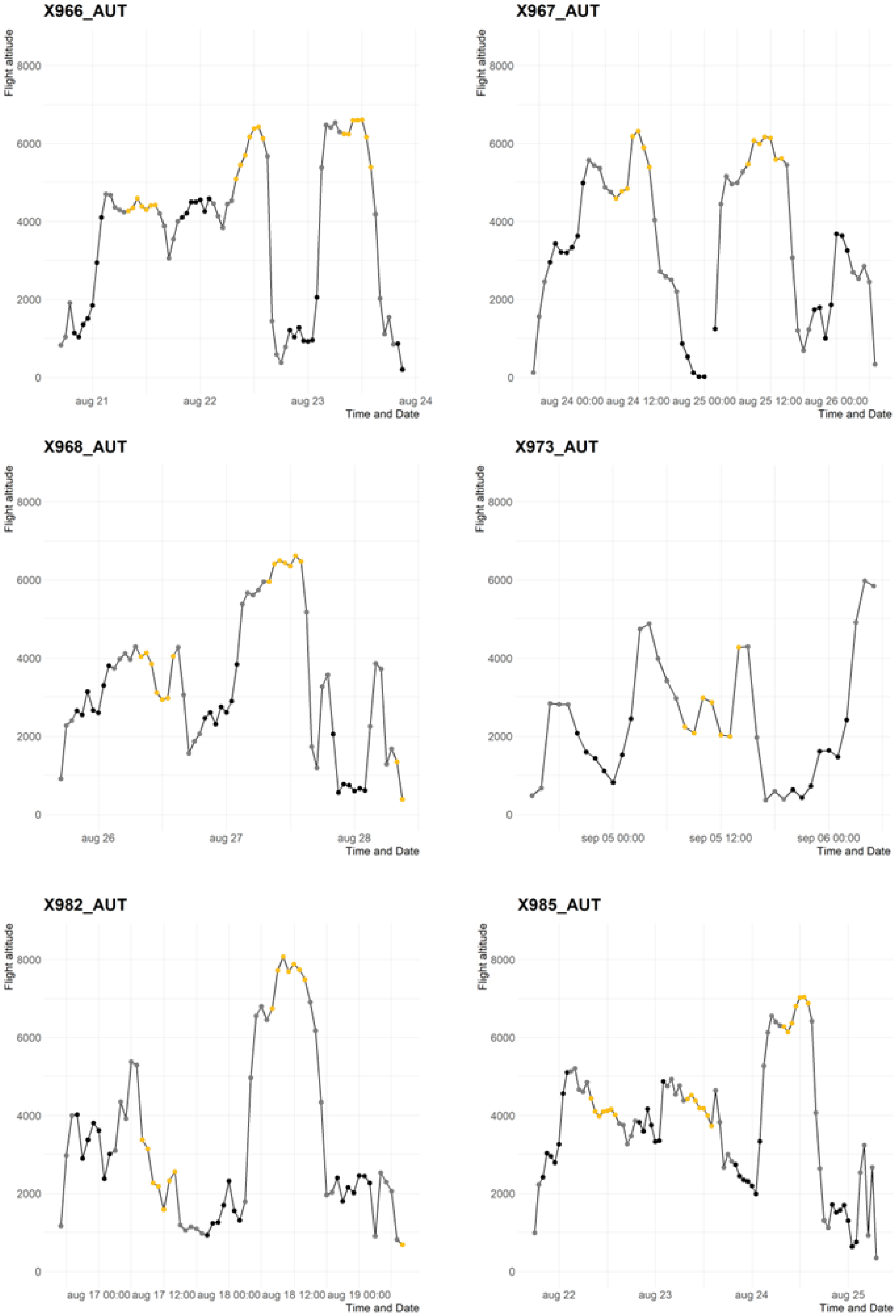
X966_AUT
Flight altitude
0
2000
4000
6000
8000
aug 21
aug 22
aug 23
aug 24
Time and Date
X967_AUT
Flight altitude
aug 24 00:00
aug 24 12:00
aug 25 00:00
aug 25 12:00
aug 26 00:00
Time and Date
X968_AUT
Flight altitude
aug 26
aug 27
aug 28
Time and Date
X973_AUT
Flight altitude
sep 05 00:00
sep 05 12:00
sep 06 00:00
Time and Date
X982_AUT
Flight altitude
aug 17 00:00
aug 17 12:00
aug 18 00:00
aug 18 12:00
aug 19 00:00
Time and Date
X985_AUT
Flight altitude
aug 22
aug 23
aug 24
aug 25
Time and Date

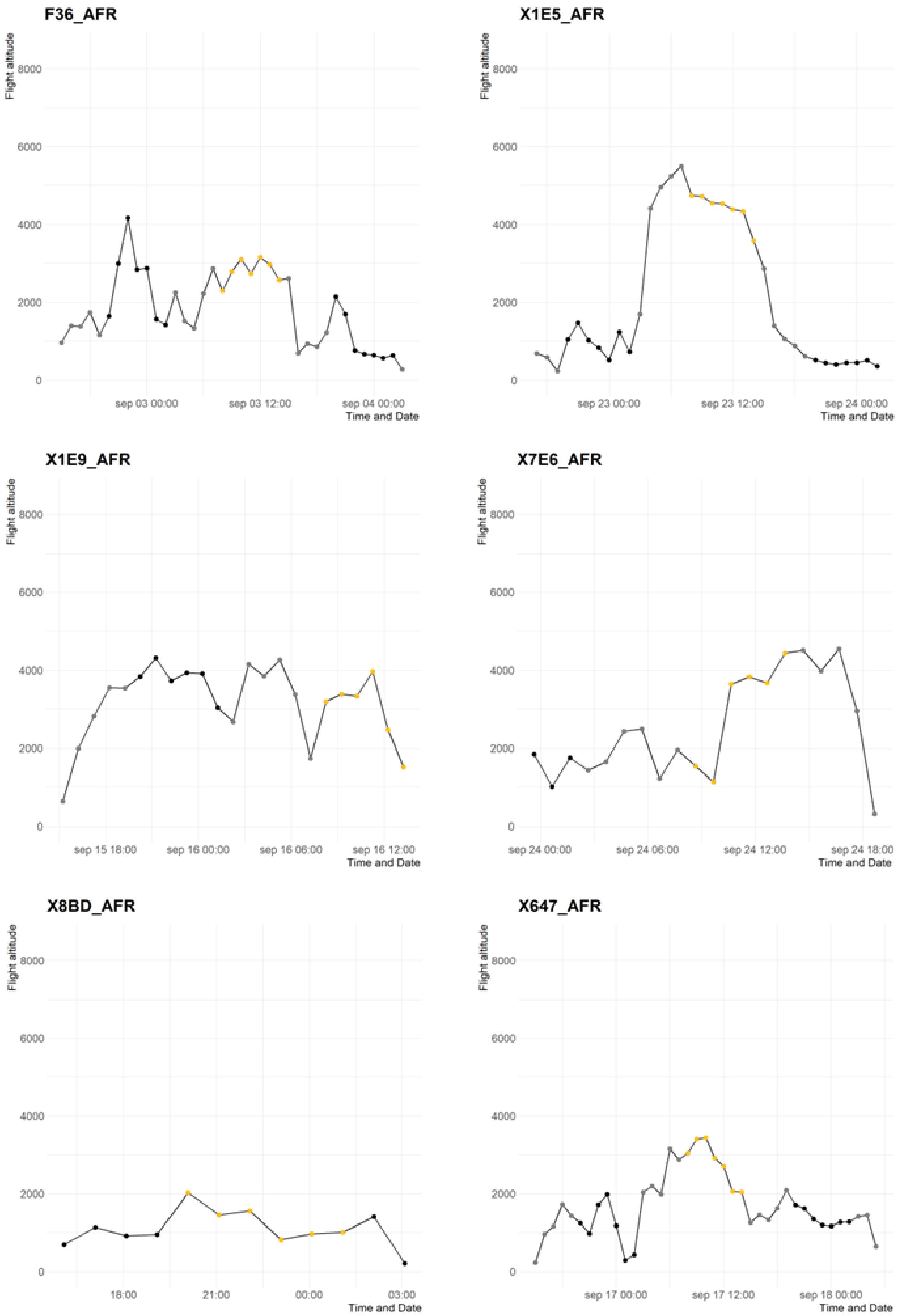
F36_AFR
Flight altitude
8000
6000
4000
2000
0
sep 03 00:00
sep 03 12:00
sep 04 00:00
Time and Date
X1E5_AFR
Flight altitude
8000
6000
4000
2000
0
sep 23 00:00
sep 23 12:00
sep 24 00:00
Time and Date
X1E9_AFR
Flight altitude
8000
6000
4000
2000
0
sep 15 18:00
sep 16 00:00
sep 16 06:00
sep 16 12:00
Time and Date
X7E6_AFR
Flight altitude
8000
6000
4000
2000
0
sep 24 00:00
sep 24 06:00
sep 24 12:00
sep 24 18:00
Time and Date
X8BD_AFR
Flight altitude
8000
6000
4000
2000
0
18:00
21:00
00:00
03:00
Time and Date
X647_AFR
Flight altitude
8000
6000
4000
2000
0
sep 17 00:00
sep 17 12:00
sep 18 00:00
Time and Date

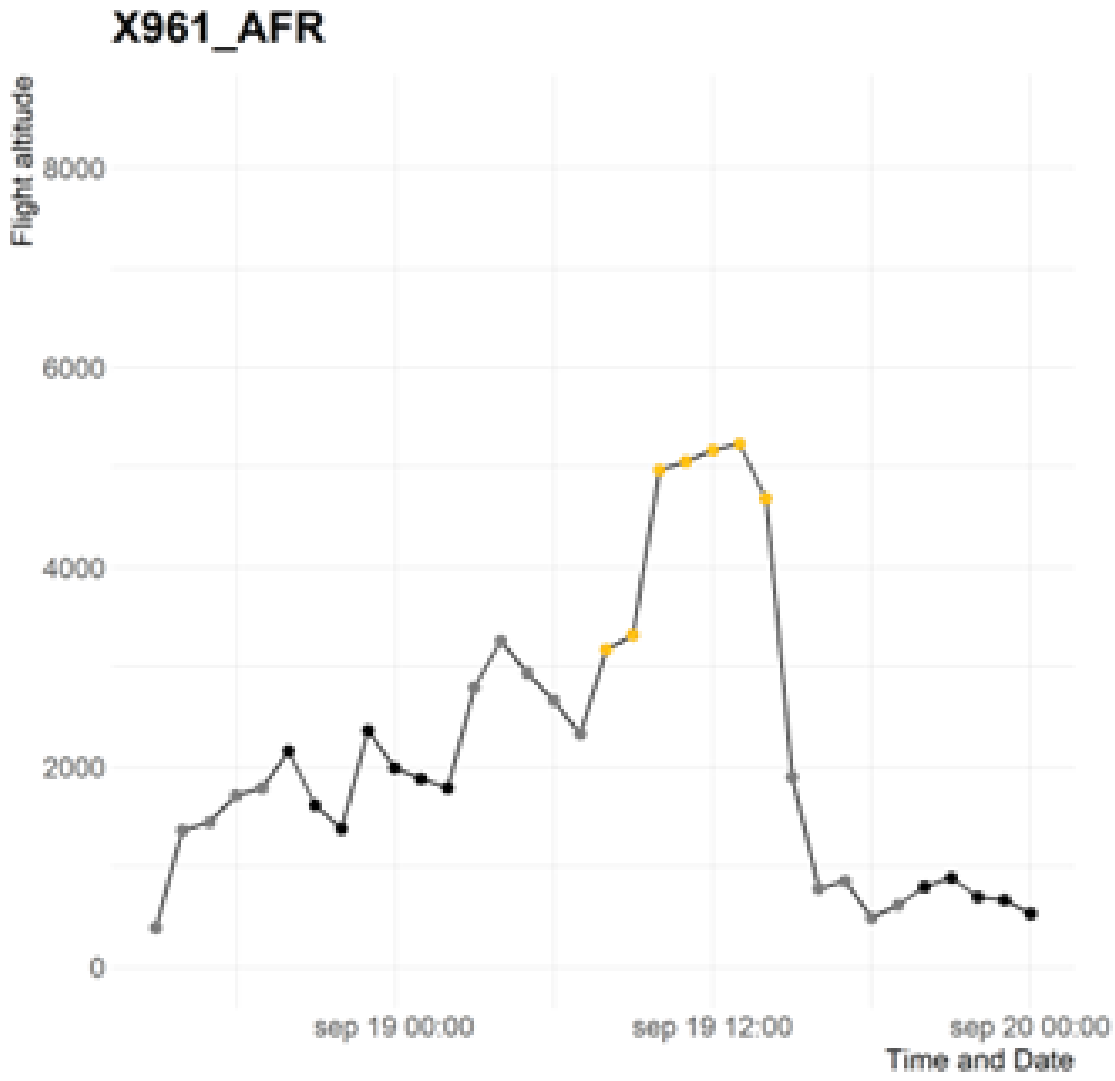
X961_AFR
Flight altitude
8000
6000
4000
2000
0
sep 19 00:00
sep 19 12:00
sep 20 00:00
Time and Date

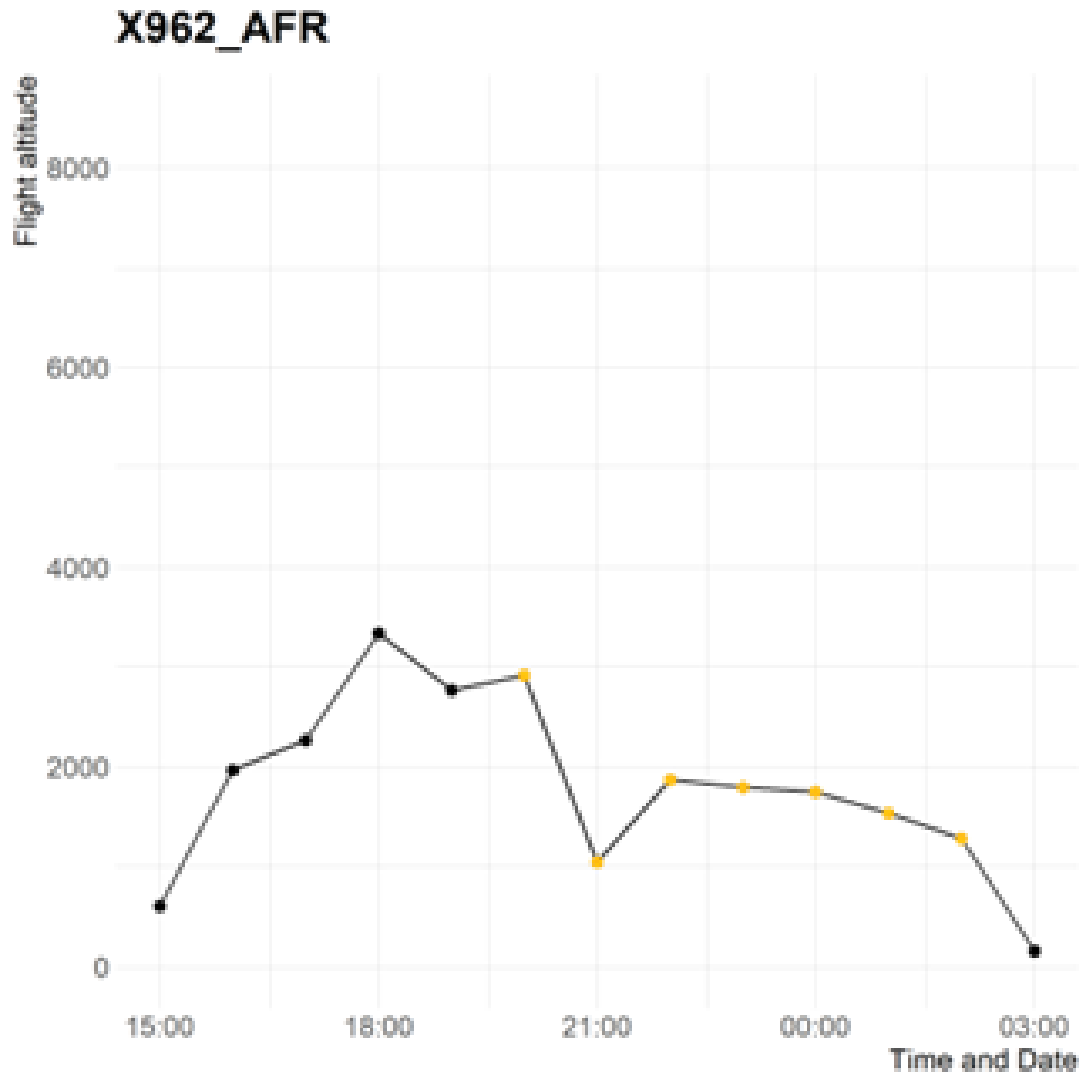
X962_AFR
Flight altitude
8000
6000
4000
2000
0
15:00
18:00
21:00
00:00
03:00
Time and Date

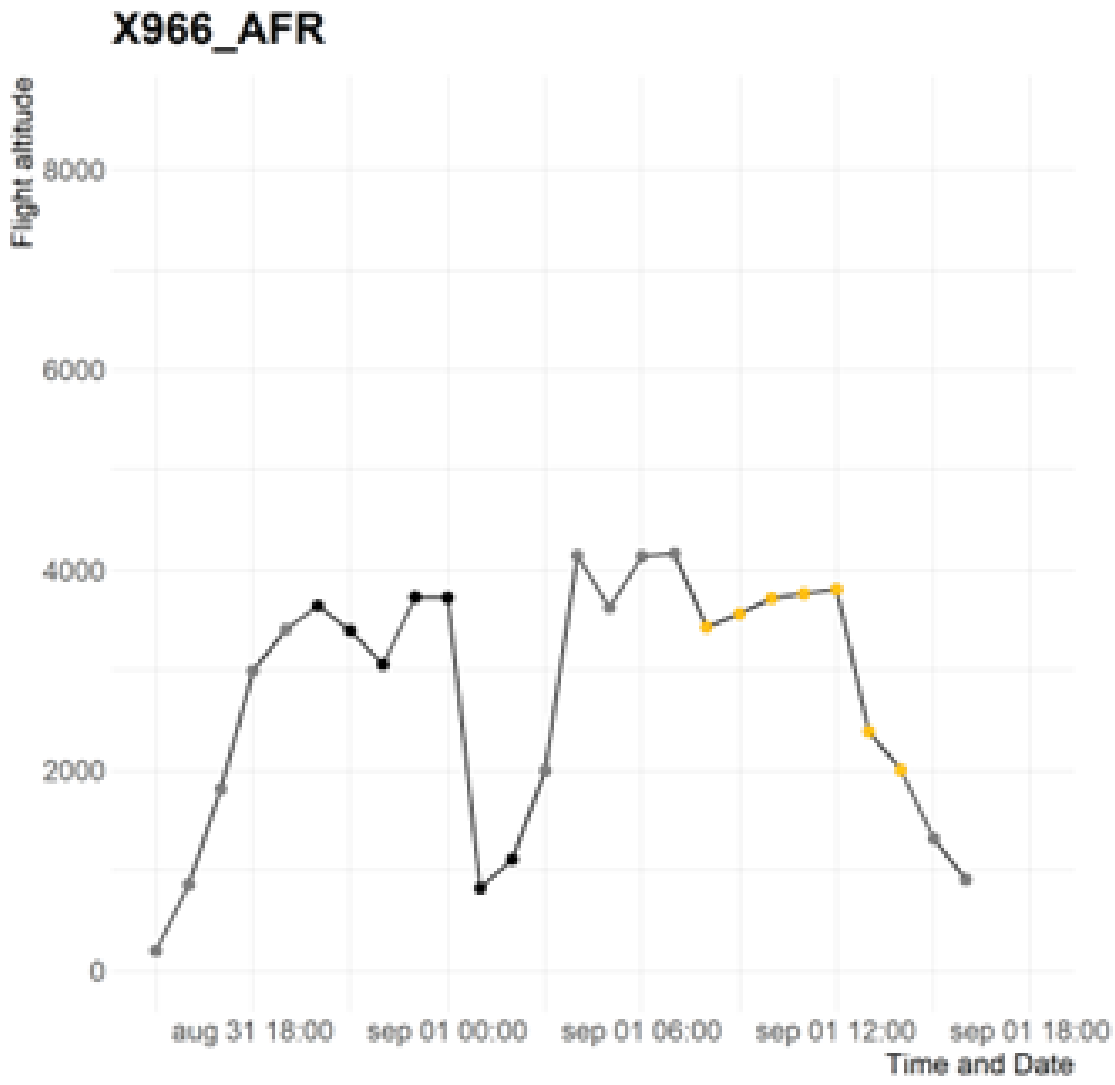
X966_AFR
Flight altitude
8000
6000
4000
2000
0
aug 31 18:00
sep 01 00:00
sep 01 06:00
sep 01 12:00
sep 01 18:00
Time and Date

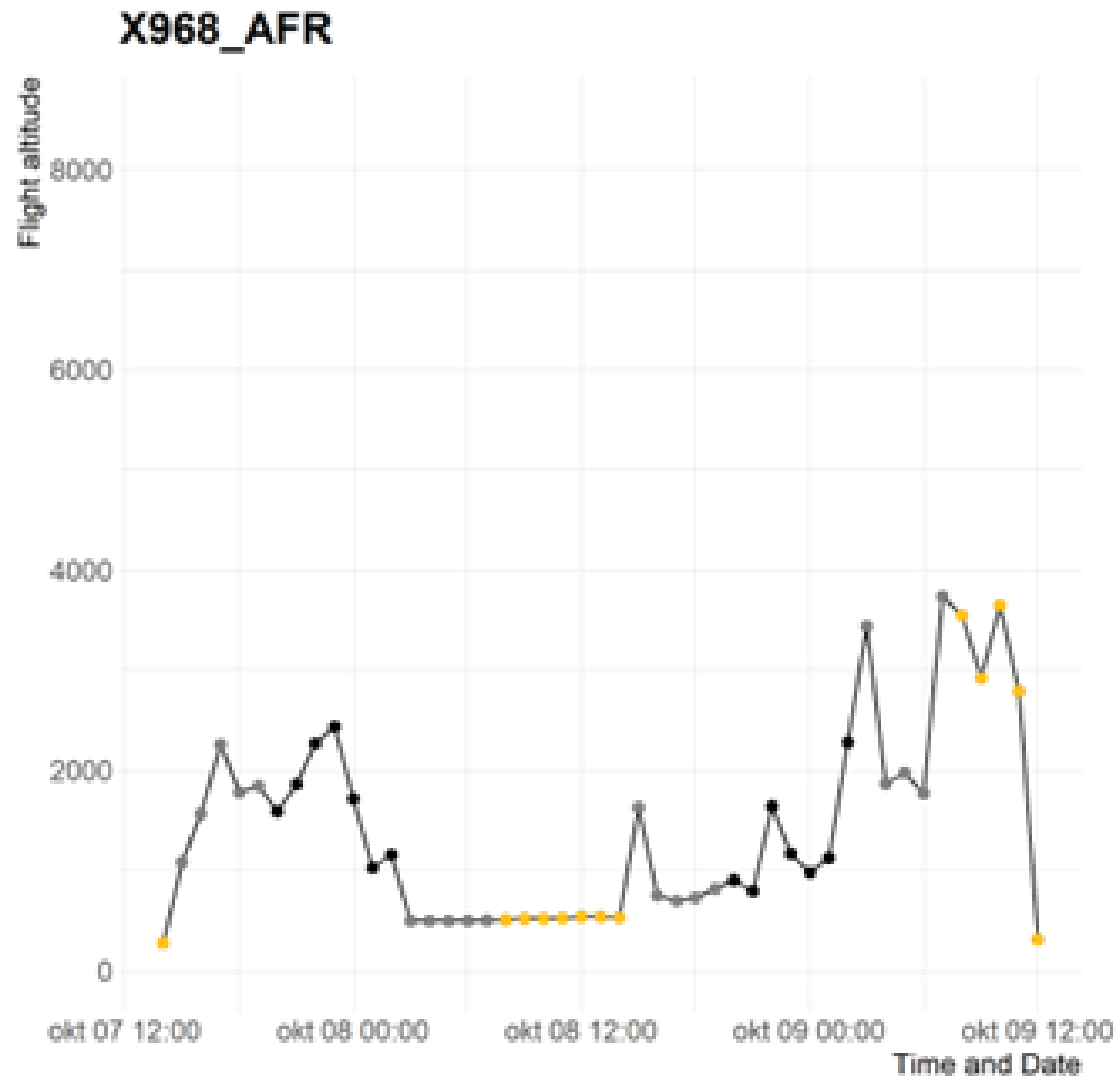
X968_AFR
Flight altitude
8000
6000
4000
2000
0
okt 07 12:00
okt 08 00:00
okt 08 12:00
okt 09 00:00
okt 09 12:00
Time and Date

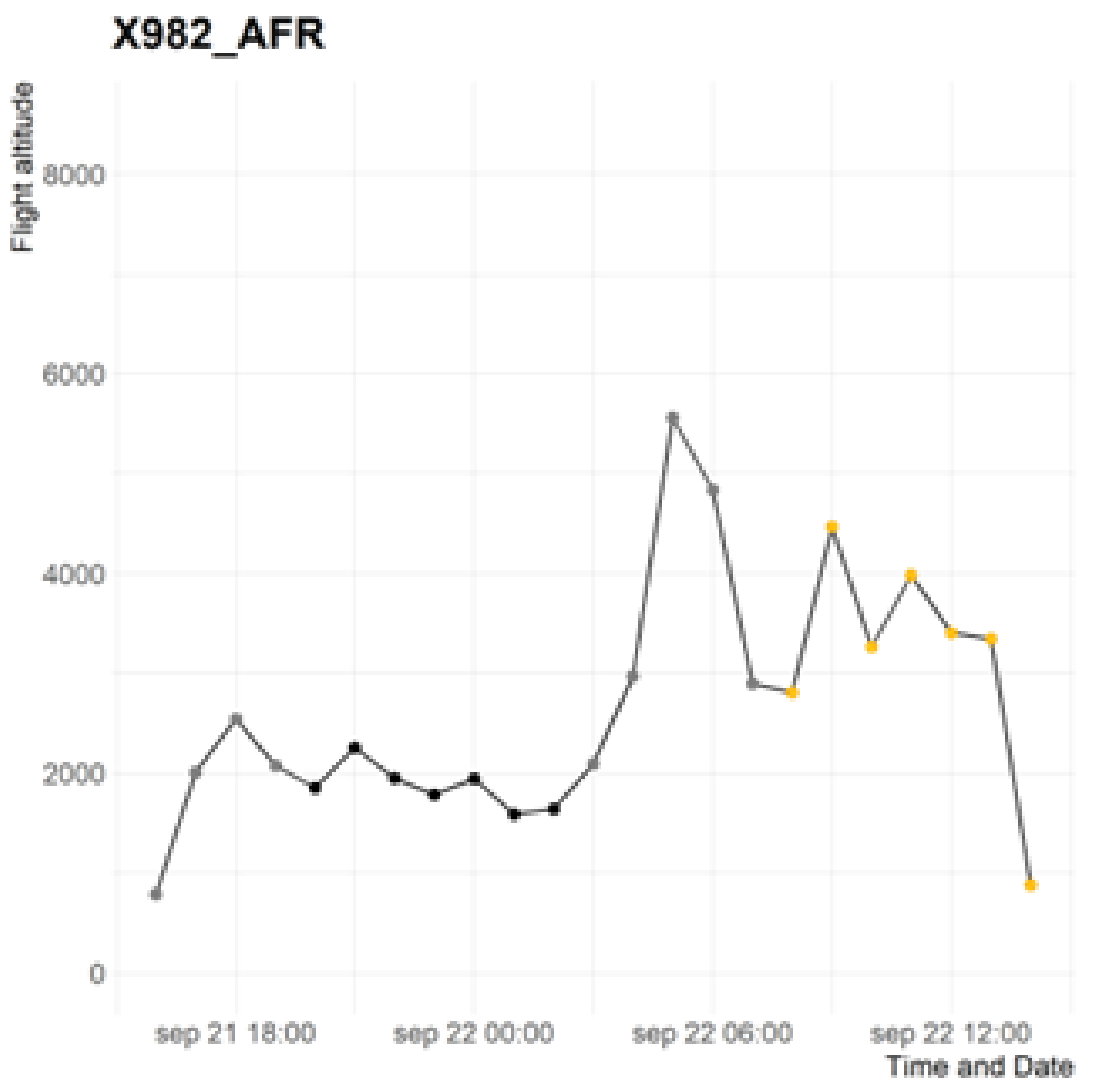
X982_AFR
Flight altitude
8000
6000
4000
2000
0
sep 21 18:00
sep 22 00:00
sep 22 06:00
sep 22 12:00
Time and Date

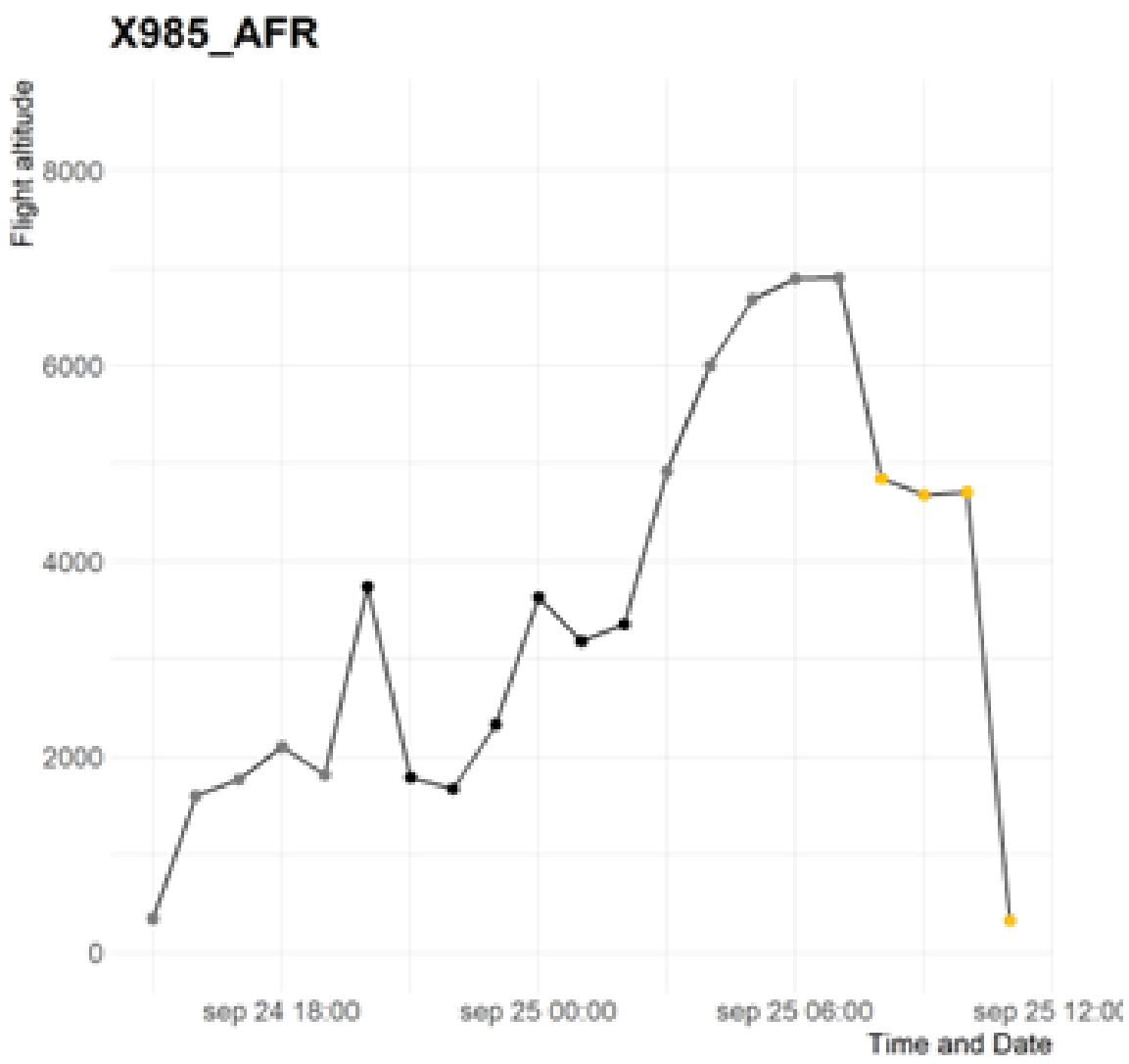
X985_AFR
Flight altitude
8000
6000
4000
2000
0
sep 24 18:00
sep 25 00:00
sep 25 06:00
Time and Date

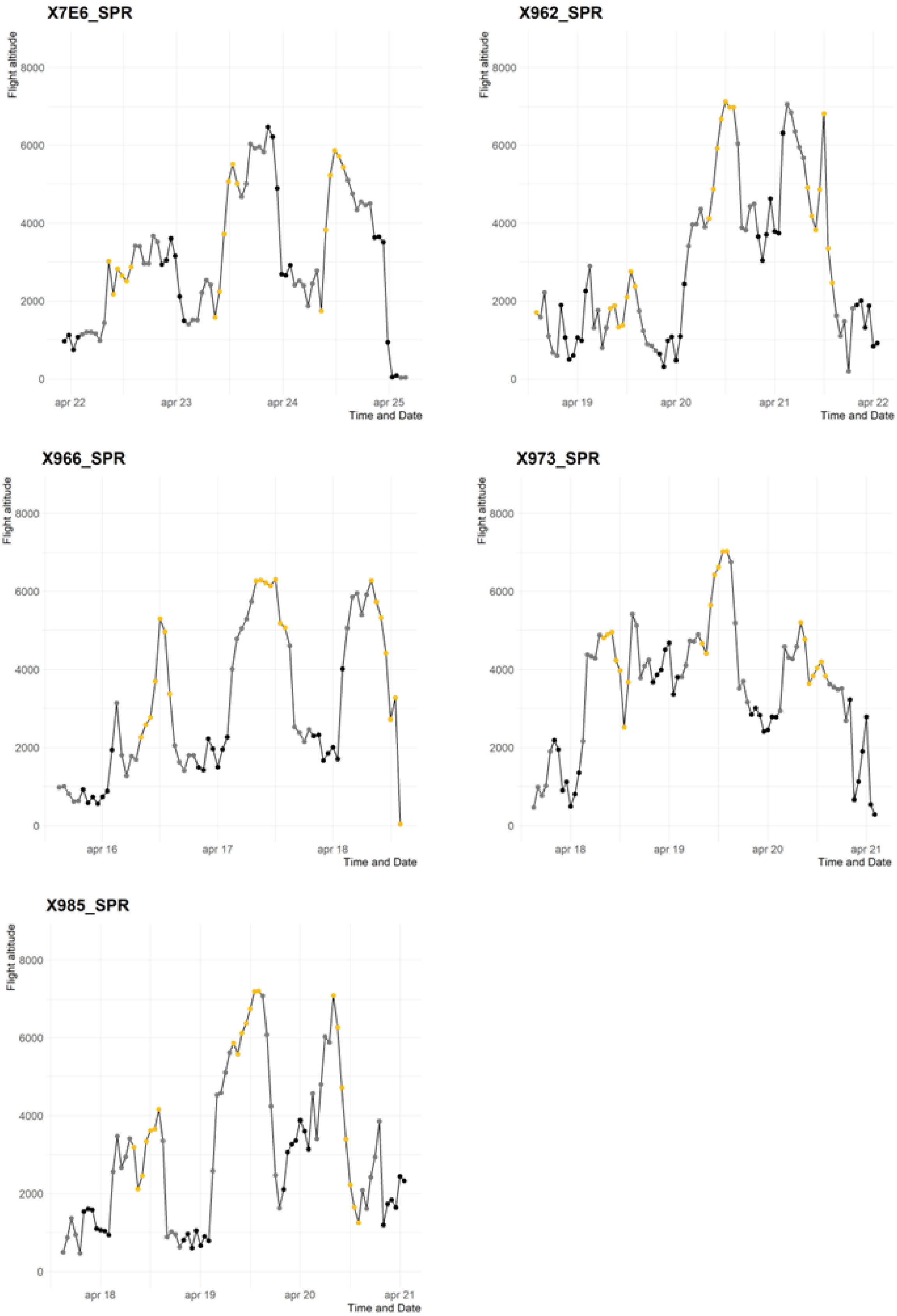

Fig. S1. Flight curves of all individual flights included in the study. Tracks with a 5-minute sampling interval have been resampled to a 1-hour temporal resolution. Certain daytime (08:00-14:00 UTC) flight altitudes are marked in yellow, certain nighttime (20:00-02:00 UTC) altitudes in black and any time in between in grey.

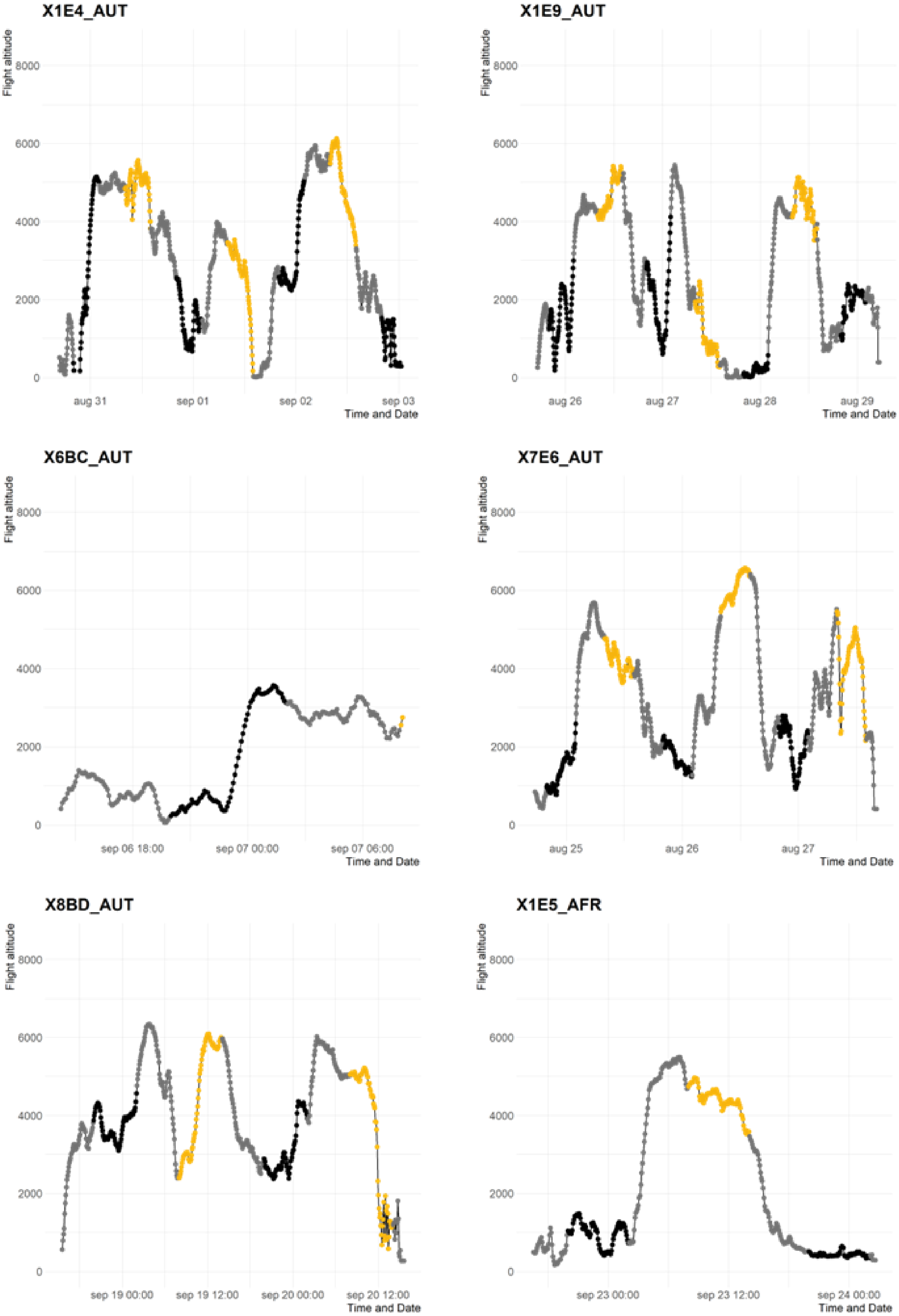

Journal of Experimental Biology • Supplementary information

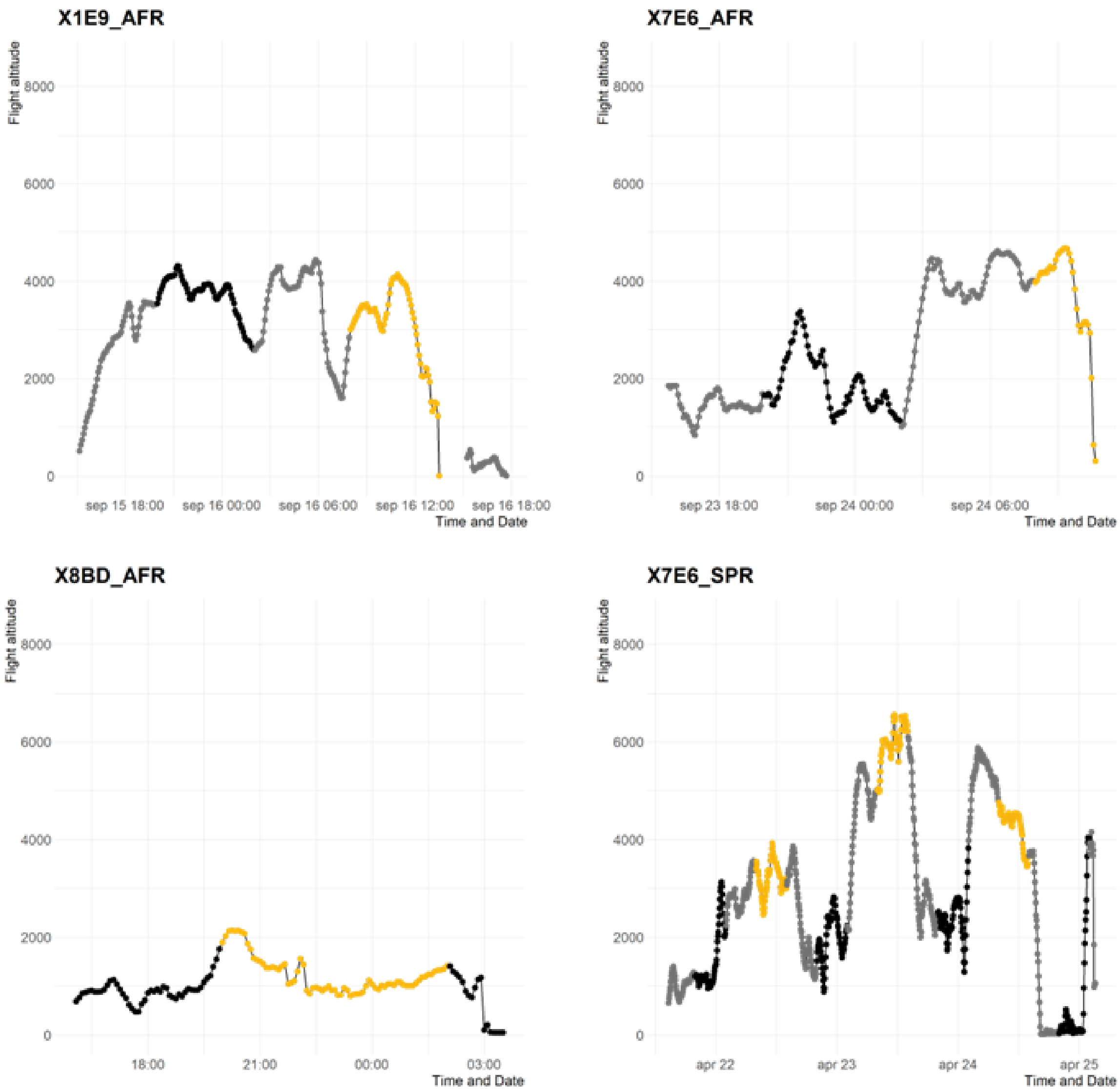

Fig. S2. Flight curves of individual flights tracked with 5-minute sampling interval. Certain daytime (08:00-14:00 UTC) flight altitudes are marked in yellow, certain nighttime (20:00-02:00 UTC) altitudes in black and any time in between in grey.

Journal of Experimental Biology • Supplementary information

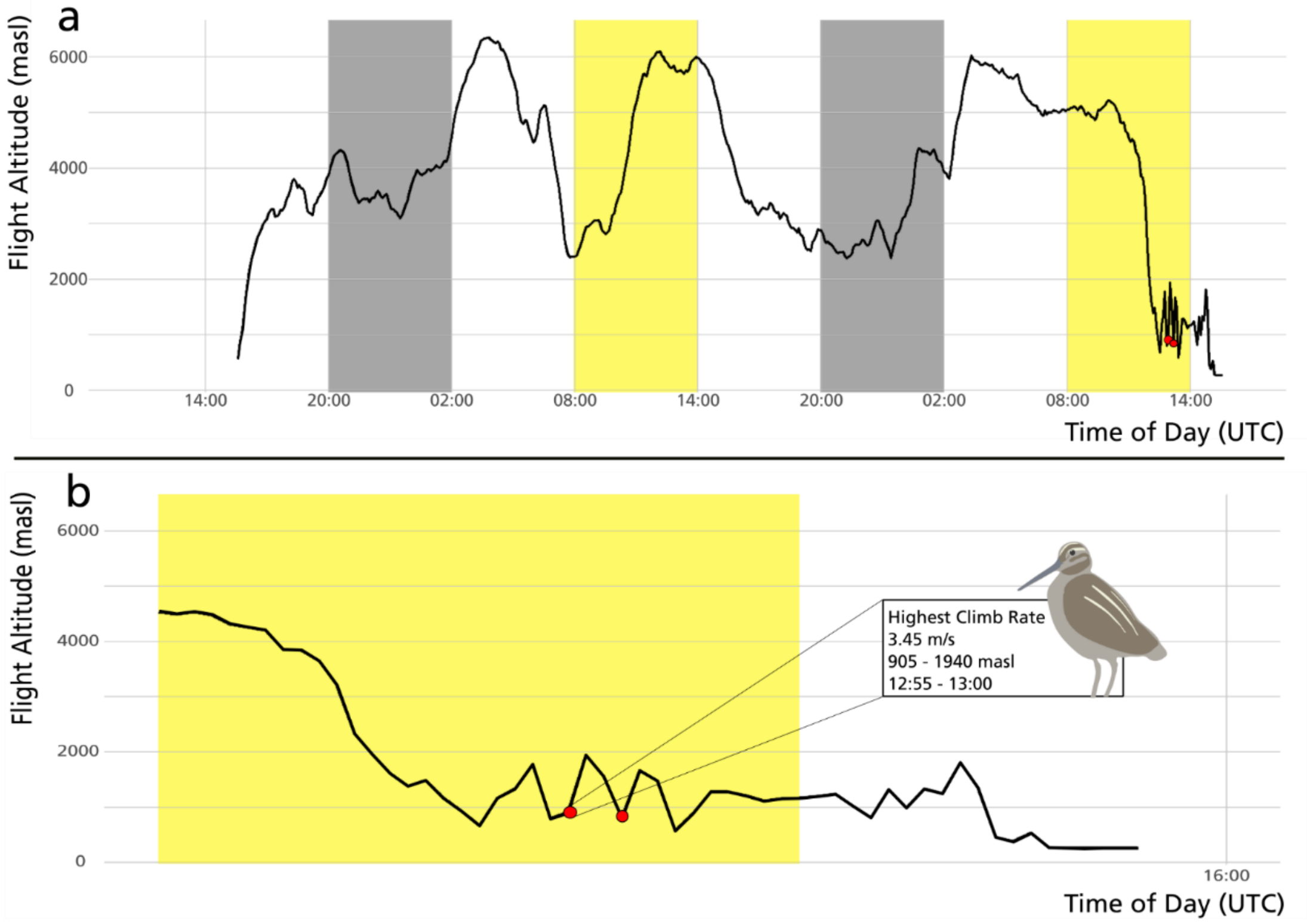

Fig. S3. a) Flight curve of the autumn flight by "8BD" with 5-minute sampling interval. Periods of certain daytime (08:00-14:00 UTC) are marked in yellow and certain nighttime (20:00-02:00 UTC) in grey. **b)** A zoomed-in view of the last part of the flight where two observations of unusually high climb rates are made, here marked in red.

Journal of Experimental Biology • Supplementary information
